# Cell Line-, Protein-, and Sialoglycosite-Specific Control of Flux-Based Sialylation in Human Breast Cells: Implications for Cancer Progression

**DOI:** 10.3389/fchem.2020.00013

**Published:** 2020-02-05

**Authors:** Christopher T. Saeui, Kyung-cho Cho, Vrinda Dharmarha, Alison V. Nairn, Melina Galizzi, Sagar R. Shah, Prateek Gowda, Marian Park, Melissa Austin, Amelia Clarke, Edward Cai, Matthew J. Buettner, Ryan Ariss, Kelley W. Moremen, Hui Zhang, Kevin J. Yarema

**Affiliations:** ^1^Department of Biomedical Engineering, Translational Tissue Engineering Center, The Johns Hopkins University, Baltimore, MD, United States; ^2^Department of Pathology, The Johns Hopkins School of Medicine, Baltimore, MD, United States; ^3^Complex Carbohydrate Research Center, University of Georgia, Athens, GA, United States; ^4^Department of Chemical and Biomolecular Engineering, Whiting School of Engineering, The Johns Hopkins University, Baltimore, MD, United States; ^5^Department of Oncology, The Johns Hopkins School of Medicine, Baltimore, MD, United States

**Keywords:** metabolic glycoengineering, ManNAc analogs, breast cancer, sialylation, sialic acid, metabolic flux

## Abstract

Sialylation, a post-translational modification that impacts the structure, activity, and longevity of glycoproteins has been thought to be controlled primarily by the expression of sialyltransferases (STs). In this report we explore the complementary impact of metabolic flux on sialylation using a glycoengineering approach. Specifically, we treated three human breast cell lines (MCF10A, T-47D, and MDA-MB-231) with 1,3,4-O-Bu_3_ManNAc, a “high flux” metabolic precursor for the sialic acid biosynthetic pathway. We then analyzed N-glycan sialylation using solid phase extraction of glycopeptides (SPEG) mass spectrometry-based proteomics under conditions that selectively captured sialic acid-containing glycopeptides, referred to as “sialoglycosites.” Gene ontology (GO) analysis showed that flux-based changes to sialylation were broadly distributed across classes of proteins in 1,3,4-O-Bu_3_ManNAc-treated cells. Only three categories of proteins, however, were “highly responsive” to flux (defined as two or more sialylation changes of 10-fold or greater). Two of these categories were cell signaling and cell adhesion, which reflect well-known roles of sialic acid in oncogenesis. A third category—protein folding chaperones—was unexpected because little precedent exists for the role of glycosylation in the activity of these proteins. The highly flux-responsive proteins were all linked to cancer but sometimes as tumor suppressors, other times as proto-oncogenes, or sometimes both depending on sialylation status. A notable aspect of our analysis of metabolically glycoengineered breast cells was decreased sialylation of a subset of glycosites, which was unexpected because of the increased intracellular levels of sialometabolite “building blocks” in the 1,3,4-O-Bu_3_ManNAc-treated cells. Sites of decreased sialylation were minor in the MCF10A (<25% of all glycosites) and T-47D (<15%) cells but dominated in the MDA-MB-231 line (~60%) suggesting that excess sialic acid could be detrimental in advanced cancer and cancer cells can evolve mechanisms to guard against hypersialylation. In summary, flux-driven changes to sialylation offer an intriguing and novel mechanism to switch between context-dependent pro- or anti-cancer activities of the several oncoproteins identified in this study. These findings illustrate how metabolic glycoengineering can uncover novel roles of sialic acid in oncogenesis.

## Introduction

Sialic acid is a unique 9-carbon sugar that caps mammalian glycans and determines many aspects of a cell's interaction with its microenvironment in health and disease (Varki, 1997, 2008; Schauer, 2009). The incorporation of this sugar into glycans generally has been assumed to be controlled primarily by STs, a family of 20 enzymes in humans (Du et al., 2009; Li and Chen, 2012). For example, early mathematical models of N-linked glycosylation (Krambeck and Betenbaugh, 2005) (including sialylation; Monica et al., 1997), were based solely on enzyme levels and activity. The idea that enzyme activity can predict glycan patterns was supported by the ability of mathematical models to be trained to reflect experimentally observed sialylation with reasonable accuracy and to predict glycosylation patterns found in different subtypes of cancers (Krambeck et al., 2009; Bennun et al., 2013). The prevailing premise that metabolic flux plays a small—perhaps even negligible role—in sialylation was consistent with the antiport nature of CMP-sialic acid import into the lumen of the Golgi where transfer of the sialic acid moiety from this nucleotide sugar donor to nascent glycoconjugates occurs. Specifically, the antiport transfer of spent CMP ***out*** of the Golgi limits flux ***into*** this organelle (Hadley et al., 2014). As a result, regardless of how much flux enters the sialic acid biosynthetic pathway via ManNAc, the committed precursor to the pathway (Keppler et al., 1999; Luchansky et al., 2004), later bottlenecks (Viswanathan et al., 2003) limit subsequent glycan sialylation. Certain experimental results support this premise, including findings that sialuria mutations of UDP-GlcNAc 2-epimerase/ManNAc kinase (GNE) that greatly increase intracellular sialic acid production (Seppala et al., 1999) do not necessarily translate into correspondingly large increases in cell surface sialylation (Yarema et al., 2001); similarly, loss-of-activity mutations do not always correspondingly diminish sialylation (Hinderlich et al., 2004; Salama et al., 2005). Finally, introduction of exogenously-supplied ManNAc (or ManNAc precursors) into cells can result in large (e.g., 10–100-fold) increases in intracellular sialic acid with minimal (e.g., only 0.05–0.25-fold) changes to surface sialylation (Jacobs et al., 2001; Jones et al., 2004).

**Figure 1:**
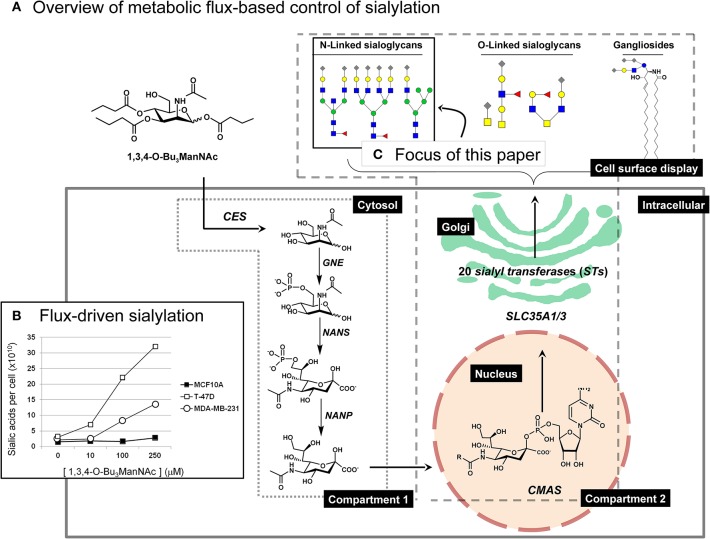
Overview of ManNAc analog-based control of flux through the sialic acid biosynthetic machinery. **(A)** The high-flux ManNAc analog 1,3,4-O-Bu_3_ManNAc efficiently diffuses across the plasma membrane after which non-specific esterases release the core ManNAc moiety by hydrolysis of the butyrate groups (Mathew et al., 2012). The kinase activity of GNE (or other kinases) and subsequent activities of NANS, and NANP in the cytosol converts ManNAc to sialic acid; these metabolites constitute “Compartment 1.” Once synthesized and dephosphorylated, sialic acid enters the nucleus where CMAS converts it to the corresponding nucleotide sugar (e.g., CMP-Neu5Ac); CMP-Neu5Ac is transported into the Golgi apparatus by SLC35A1 and SLC35A3 where a subset (depending on the cell line) of the 20 human STs create sialoglycoconjugates (primarily, N- and O-linked glycoproteins or gangliosides [i.e., sialic acid-modified glycosphingolipids]). **(B)** Representative results from a previous study (Saeui et al., 2018) are shown to illustrate the minimal (in the MCF-10A line) to large increase (in the T-47D line) that occurs in flux-driven sialylation in human breast cell lines (note that statistical significance for similar results is provided in Figure 2). **(C)** The focus of the current paper is to conduct a detailed “glycosite” analysis of cell surface glycoproteins obtained from 1,3,4-O-Bu_3_ManNAc treated cells. This figure is adapted from our previous publication (Saeui et al., 2018).

Gaining clear-cut evidence for flux-based changes to sialic acid has been hampered by technical difficulties in introducing ManNAc, the precursor for sialic acid biosynthesis (Luchansky et al., 2003), into cells. Mammalian cells lack plasma membrane transporters for this sugar, necessitating uptake by pinocytosis. As a consequence, internalization is not saturated even at very high concentrations of exogenous ManNAc (e.g., 75 mM; Yarema et al., 1998), at which point osmotic stress decreases cell viability and adversely affects sialylation. Similar pitfalls—decreased cellular viability and even overt cytotoxicity (Jones et al., 2004; Kim et al., 2004a,b)—occurs with peracetylated sugar analogs (Sarkar et al., 1995, 1997). For context, peracetylation is a strategy that facilitates cellular uptake of ManNAc (Hadfield et al., 1983; Schwartz et al., 1983; Lemieux et al., 1999). Once peracetylated ManNAc is inside a cell, non-specific esterases (Mathew et al., 2012, 2017) remove the ester-linked acetate groups or other short chain fatty acids (SCFAs) such as propionate or butyrate (Kim et al., 2004b; Sampathkumar et al., 2006; Hao et al., 2019). Our team overcame these difficulties by omission of ester-linked SCFAs from the C6-OH position of ManNAc, which largely eliminates cytotoxicity (Aich et al., 2008; Wang et al., 2009) and ameliorates other off-target effects (Campbell et al., 2008; Elmouelhi et al., 2009). By using the resulting “high flux” analogs (exemplified by 1,3,4-O-Bu_3_ManNAc, Figure 1) we can introduce saturating levels of flux into the sialic acid pathway at sub-cytotoxic levels (Almaraz et al., 2012a; Yin et al., 2017, 2018).

Having developed 1,3,4-O-Bu_3_ManNAc to effectively enhance sialylation, we exploited this glycoengineering tool to gain insight into the role of sialic acid in cancer progression. For example, we previously used 1,3,4-O-Bu_3_ManNAc to increase sialylation in SW1990 pancreatic cancer cells and determined changes in N-glycan sialylation through glycosite analysis (Almaraz et al., 2012b; Shah et al., 2013; Tian et al., 2015). In parallel we linked these changes to cell behaviors associated with cancer such as integrin-mediated cell motility (Almaraz et al., 2012b) and EGFR-related drug sensitivity (Mathew et al., 2015, 2016). Although limited to a single cell line, these results unambiguously showed that metabolic flux can influence sialylation; cell surface sialic acid increased globally by ~75% and individual glycosites increased by as much as ~8-fold. Subsequent studies expanded our analyses to multiple cell lines and to a different type of cancer by comparing intracellular sialic acid production in different subtypes of breast cancer (Saeui et al., 2018). Certain results from the breast cell lines were consistent with the known role of sialic acid in cancer; for example, flux-driven sialometabolites increased dramatically in the T-47D breast cancer line compared to a much smaller increase in the near-normal MCF10A line (Figure 1), consistent with the well-known oncogenic role of sialic acid. By comparison, sialic acid production in the advanced triple negative MDA-MB-231 line was lower than in the early stage T-47D line; this finding was unexpected because oncogenesis is driven by sialic acid leading to the presumption that advanced cancers have higher levels of this sugar.

In the current study, we sought additional insight into flux-driven sialylation at various stages of breast cancer by conducting glycosite evaluation of sialoglycans of the MCF10A, T-47D, and MD-MD-231 lines using solid phase extraction of glycopeptide (SPEG) analysis (Zhang et al., 2003; Tian et al., 2007). All together, 1,410 sites of N-linked glycosylation were identified in common across these three breast cell types. As described in this report, the newly-obtained results raise intriguing new insights into the role of metabolic flux-based control of sialic acid in oncogenesis.

## Materials and Methods

### Materials

We purchased chemical reagents from Sigma-Aldrich (St. Louis, MO) to synthesize, purify, and characterize 1,3,4-O-Bu_3_ManNAc as previously described (Aich et al., 2008); characterization data is provided in Supplemental File S1. We purchased the cell lines MCF10A (ATCC CRL-10317), T-47D (ATCC HTB-133), and MDA-MB-231 (ATCC CRM-HTB-26) from the American Type Culture Collection (ATCC, Manassas, VA). Cell lines were authenticated by the Johns Hopkins Genetic Resources Core Facility using short tandem repeat (STR) profiling according to the National Institutes of Health (NIH) recommendations and by cross-referencing the resulting STR data with both the ATCC and the German Collection of Microorganisms and Cell Cultures (DSMZ) data repositories for cell authentication.

### Cell Culture

We maintained stock cultures of the T-47D and MDA-MB-231 cell lines in RPMI-1640 medium (Corning 10-040-CV) supplemented with 10% fetal bovine serum (v/v) (Corning 35-011-CV) and the appropriate dilution of 20× antibiotic-antimycotic solution (Thermo Fisher Scientific 15240062). MCF10A cells were maintained in the same media supplemented with 10 μg/mL insulin (Thermo Fisher Scientific 12585014) and 5.0 μg/mL hydrocortisone (Sigma Aldrich H0888); these media are referred to as “growth media” below. As noted below, sialic acid metabolism and quantification experiments were conducted with 1.0% fetal bovine serum (v/v) in the absence of antibiotic-antimycotic solution to avoid ST inhibition and to reduce the salvage and recycling of sialic acid-containing serum components (Bonay et al., 1996; Badr et al., 2015a,b); this low serum antibiotics-free medium is referred to as “assay media” below. We also note that hydrocortisone can increase sialylation (e.g., in HeLa (Carubelli and Griffin, 1967) and Chinese hamster ovary (Rouiller et al., 2012) cells); it is unknown if it affects MCF10A cells in this way but we do not believe this to be a significant confounding factor in the current set of experiments because this reagent was included in both untreated control cells and 1,3,4-O-Bu_3_ManNAc-treated test cells.

### Cell Proliferation and Sialic Acid Production Assays

Cells cultured in growth media were collected via trypsinization, counted, and plated in 150 mm tissue culture dishes (5.0 × 10^6^ cells per dish) in 20 mL of assay medium. The cells were allowed to attach to the plates overnight, and then treated with 1,3,4-O-Bu_3_ManNAc (typical concentrations tested include 0, 10, 50, 100, and 250 μM, exact concentrations used in any particular experiment are indicated below). Appropriate dilutions of analog were added to each test condition from a 100 mM stock solution (stock solutions were either maintained in ethanol and stored at −20°C for up to 3 months or as lyophilized analog, which is stable when stored under nitrogen at −80°C for up to 2 years). The untreated controls were exposed to the equivalent volume of ethanol given to cells subject to the 250 μM dose (the maximum amount of ethanol added [0.25% v/v] has previously been shown to have no observable effect on cell growth, viability, or sialylation). At the specified time points (6, 24, and 48 h), the cells were detached using non-enzymatic buffer (Cellstripper Corning 25-056-CI) and cell counts were performed as previously described (Almaraz et al., 2012a) using a Beckman-Coulter Z2 Coulter Counter. Each experiment was performed in triplicate and cell counts were normalized to untreated controls. Intracellular free and conjugate bound sialic acid levels were determined for treated (100 μM) and untreated cells using the periodate resorcinol method as previously described (Jourdian et al., 1971; Yarema et al., 2001; Saeui et al., 2018).

### Transcript Analysis of Sialic Acid Metabolism and Glycosylation (SAMG) Genes

Cells were treated with 1,3,4-O-Bu_3_ManNAc at 0 or 100 μM in assay media using 100 × 20 mm tissue culture plates and 3.0 × 10^6^ cells per dish. After incubation with the analogs for 24 h, the cells were harvested by scraping, counted, and portioned into aliquots of 1.0 × 10^6^ cells that were flash-frozen in liquid nitrogen and stored at −80°C until analysis. Total RNA isolation and cDNA synthesis on three biological replicates of each treatment condition was carried out as described previously for quantitative RT-PCR analysis of SAMG genes; e.g., sialyltransferases and Golgi transporters (Nairn et al., 2007). The qRT-PCR reactions were performed in triplicate for each gene analyzed using primer pairs listed in Supplemental File S2. Amplification conditions and data analysis was performed as described (Nairn et al., 2010; Saeui et al., 2018); briefly, Ct values for each gene were normalized with the control gene, *RPL4*, prior to calculation of relative transcript abundance. Each experiment and PCR analysis was performed in triplicate. Statistical analyses were conducted for pairs of samples as well as multiple sample and treatment comparisons (Tukey's test).

### SPEG Analysis of Sialoglycopeptide

Cells were incubated with 0 or 100 μM 1,3,4-O-Bu_3_ManNAc for 24 h in assay media (eight 150 × 25 mm plates were used for each condition to obtain ≥10^9^ cells per condition at the end of the incubation period). After treatment cells from each condition were pooled and fractions were subjected to modified SPEG analyses (Zhang et al., 2003; Tian et al., 2007). Briefly, each pooled sample was subject to trypsin digestion using 1.0 mg of protein; the resulting peptides were separated by C18 chromatography using 60% acetonitrile with 0.1% TFA; and 200 μL of the C18 eluate was used for protein enrichment. Of note, we used a modification to the originally reported SPEG procedure to selectively oxidize sialic acids by using 1.0 mM precooled sodium periodate for 15 min, as reported in Almaraz et al. (2012b). These mild conditions avoid non-specific glycan oxidation, resulting in selective capture and identification of sialylated glycopeptides, which are termed “sialoglycosites” throughout this report.

## Results

### Cell Viability and Sialic Acid Metabolism

**Figure 2:**
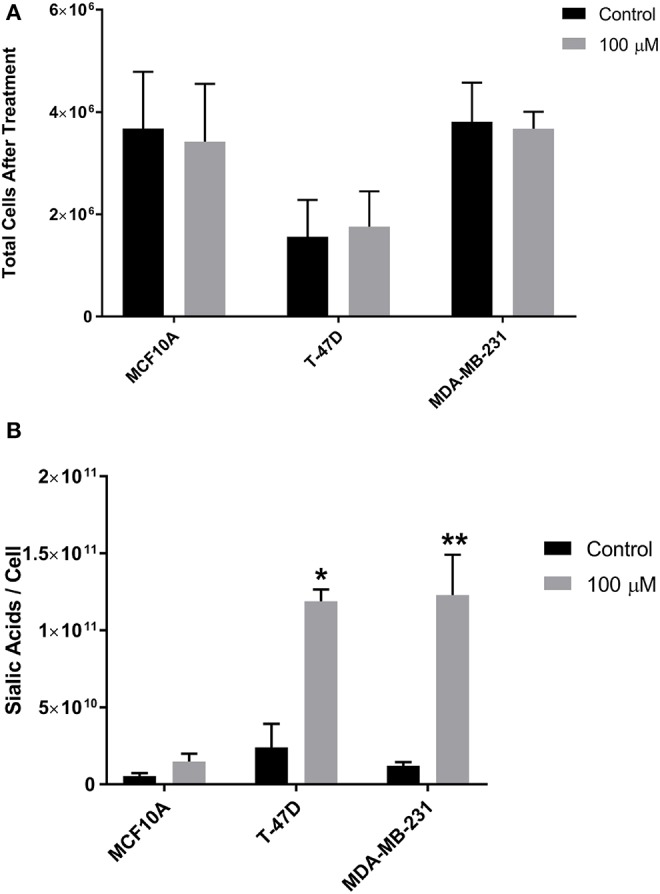
Growth rate (cell viability) and sialic acid production. **(A)** Cells were incubated with 1,3,4-O-Bu_3_ManNAc (or with solvent vehicle as a control) and counted after 48 h. **(B)** Aliquots of the cells were subject to the periodate resorcinol assay to determine sialic acid levels. Periodate-resorcinol assays were performed in triplicate. Significance was assessed using Student's *t*- test, **p < 0.01, *p < 0.05. Error bars indicate ± SEM.

The impact of 1,3,4-O-Bu_3_ManNAc on intracellular sialic acid metabolism was evaluated using conditions (e.g., 100 μM treatment for 24 h; Almaraz et al., 2012a; Saeui et al., 2018) we previously optimized across several human cell lines to provide a substantial increase in flux into the sialic acid biosynthetic pathway (Almaraz et al., 2012b) while avoiding decreased cell viability (Jones et al., 2004; Kim et al., 2004b). ManNAc analog treatment did not affect cell counts in any of the three breast cell lines compared to untreated controls (Figure 2A). In this study we used cell counts as a surrogate measure for cell viability; we have reported more extensive and rigorous evaluation of growth inhibition and cytotoxicity elsewhere (Jones et al., 2004; Kim et al., 2004b; Sampathkumar et al., 2006; Almaraz et al., 2012a). Sialic acid levels increased in all lines, although without statistical significance in the near-normal MCF10A line despite almost doubled levels in these cells (Figure 2B). These results were consistent with a previous study where we documented increased flux into the sialic acid biosynthetic pathway upon 1,3,4-O-Bu_3_ManNAc-supplementation (Saeui et al., 2018). After confirming that 1,3,4-O-Bu_3_ManNAc had the expected impact on intracellular sialic acid metabolism, we conducted detailed glycoproteomics characterization (as described below) to evaluate its downstream impact on sialoglycoconjugate formation.

### Pearson Correlation and Hierarchal Clustering

**Figure 3:**
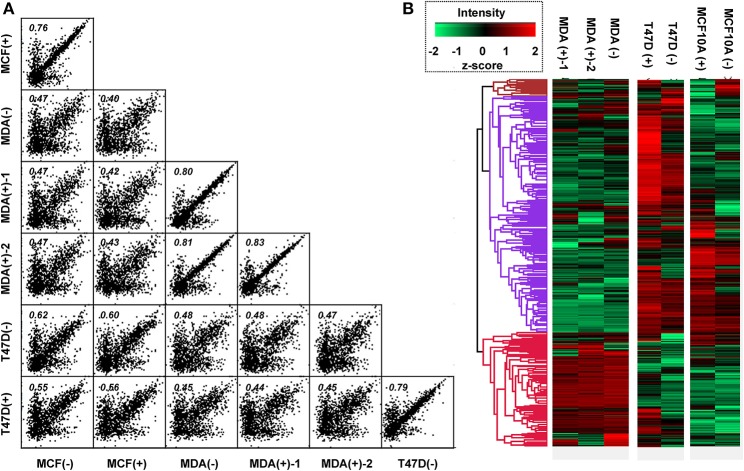
Pearson's correlation and hierarchical heatmap clustering of SPEG results. **(A)** Each cell line and condition was compared with each other to calculate a Pearson's Correlation coefficient. **(B)** A hierarchical heatmap was generated to provide global insight into the changes in and between the sialoproteomes for each type of cell upon treatment with 1,3,4-O-Bu_3_ManNAc. The (+) notation indicates samples treated with 100 μM of 1,3,4-O-Bu_3_ManNAc while (–) indicates untreated controls. Duplicate evaluation (independent biological replicates) of the MDA-MB-231 line is indicated with “-1” and “-2” [e.g., MDA(+)-1 and MDA(+)-2].

In the next experiments, we conducted Pearson correlation and hierarchal clustering after SPEG isolation and identification of the sialylated glycopeptides. We found that the quantification of sialylated peptide abundance had an acceptable Pearson correlation coefficient (*r*) of approximately 0.8 between the same cell lines as well as in replicate runs whereas the *r* value between cell lines was under 0.62 (MCF10A vs. T-47D) or 0.5 (MCF10A vs. MDA-MB-231 or T-47D vs. MDA-MB-231) (Figure 3A). Furthermore, the abundance of sialylated peptides in each cell line showed definite hierarchal clustering tendencies, regardless of 1,3,4-O-Bu_3_ManNAc treatment (Figure 3B). An interesting facet of the hierarchal clustering was that the T-47D cancer line showed greater similarity to the near-normal MCF10A line than to the advanced stage MDA-MB-231 cancer cells. For example, peptides over-represented in the MDA-MB-231 cells, which appear near the bottom of the heatmap, were under-represented in the other two lines, and *vice versa*. This result presaged subsequent results where the sialylation characteristics uncovered by our glycoengineering approach diverged substantially for the advanced MDA-MB-231 line compared to the MCF10A and T-47D lines.

Hierarchal clustering data also showed that 1,3,4-O-Bu_3_ManNAc altered sialylglycopeptide abundance ***within*** a cell type (Figure 3B) as evident when comparing control “(−)” and analog-treated “(+)” sample heatmaps from each cell line. In general, the MCF10A and T-47D cell lines responded to 1,3,4-O-Bu_3_ManNAc treatment as expected with an unambiguous overall increase in sialylation for many of the sialoglycopeptides, as indicated in red. Furhtermore, increased sialylation was more pronounced in T-47D cells compared to the MCF10A line. The response to 1,3,4-O-Bu_3_ManNAc observed in the MCF10a and T-47D lines was consistent with the generally accepted premise that cancer cells (i.e., the T-47D line) have greater sialylation compared to normal cells (represented by the near-normal MCF10A line). Hierarchal clustering analysis of the MDA-MB-231 line, by contrast, did not show any clear trend toward increased sialylation upon 1,3,4-O-Bu_3_ManNAc treatment, which was puzzling considering the generally accepted role of sialic acid in cancer progression.

### Flux-Based Control of Glycosite Sialyation

#### Flux-Based Regulation of Sialoglycosites—Global Considerations

**Figure 4:**
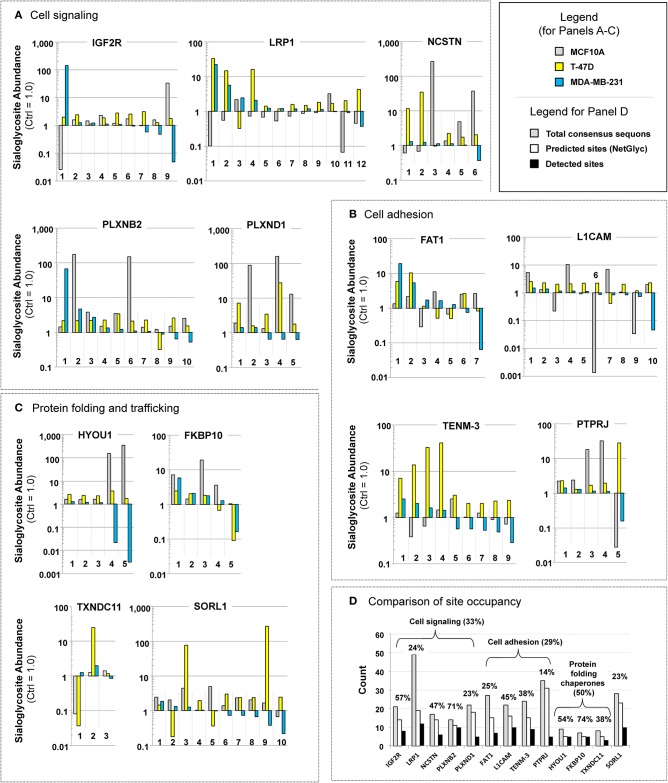
Examples of proteins with “glycosites” with large increases (up or down or both) in sialylation. Proteins are shown that illustrate how flux-driven sialylation can selectively influence glycopeptide sialylation at the cell, protein, or sub-protein (i.e., sialoglycosite) levels that fall into three functional categories: **(A)** cell signaling, **(B)** cell adhesion, and **(C)** protein folding chaperones. Note that in this figure each sialoglycosite is arbitrarily numbered, the exact sites within each protein are provided in Supplemental File S5. **(D)** The absolute number of consensus sequons (gray bars), predicted sites of N-glycans (white bars, as predicted by NetNGly), and observed sites of sialylglycopeptides identified in this study for each of the eight highly responsive oncoproteins listed in **(A–C)**. The percentages listed represent the number of experimentally observed compared to the total predicted number of N-glycan consensus sequon sites.

The SPEG analysis identified 1410 sialylated glycopeptides that were present in all three breast cell lines (the complete data set is supplied in Supplemental File S3). To understand the biological significance of this data set, we conducted GO analysis of these 1410 glycosites and found they were spread across many functional categories (Supplemental File S4) and included both oncogenic proteins and those not related to cancer. Because we did not gain meaningful insights from this global data analysis, we next focused on proteins where at least two changes in sialoglycosite sialylation of >10-fold occurred upon 1,3,4-O-Bu_3_ManAc treatment. In some cases these changes occurred at different sialoglycosites in the same cell line, in other cases the changes occurred at the same sialoglycosite in different lines, while in other cases the changes occurred at different sialoglycosites in different lines. In the selection of these proteins, we counted both increases and decreases in sialylation of >10-fold and found 13 proteins that met these criteria (Figure 4; of these proteins three had two sialoglycosites with >10-fold changes, three proteins had three such sialoglycosites, six proteins had four, and one had five).

A case-by-case evaluation of these proteins—which we deem to be “highly flux-responsive” because of these large changes (i.e., at least two changes of 10-fold or more) in sialoglycosite abundance upon treatment with 1,3,4-O-Bu_3_ManNAc—revealed interesting biological features. First, in contrast to the global GO analysis, the highly flux-responsive proteins disproportionately fell into two expected categories as well as an unexpected category. The first category includes proteins involved in cell signaling (Figure 4A), a second category includes proteins involved in cell adhesion (Figure 4B), and the third (unexpected) category consists of chaperones linked to protein folding (Figure 4C). One notable aspect of these findings was that proteins involved in neural development were strongly represented (i.e., five of the 13 [LRP1, PLXNB2, PLXND1, L1CAM, and TENM3] fit into this category). We discuss each of these categories in more detail next including the cancer-relevance of each of the 13 proteins.

#### Cell Signaling (Figure 4A)

Sialic acid is strongly linked to cell signaling (Allende and Proia, 2002; Schauer, 2009; Parker and Kohler, 2011); accordingly, we were not surprised that proteins identified as highly responsive to metabolic flux fell into this category. One of these, **IGF2R** (insulin-like growth factor 2 receptor), is a dual receptor for insulin-like growth factor 2 and mannose 6-phosphate. Functions of IGF2R include intracellular trafficking of lysosomal enzymes, activation of TGFβ, and the degradation of IGF2 (Bergman et al., 2013). A regulatory circuit links the insulin/IGF system with cancer through the glycosylation status of IGF2R (de-Freitas-Junior et al., 2017); in one example, desialylation of insulin receptors controls the proliferation of L6 myoblasts (Arabkhari et al., 2010). Finally, human tumors (including breast carcinomas) show genetic loss or mutation of IGF2R (Kalla Singh et al., 2010). A second receptor, **LRP1** (low-density lipoprotein receptor-related protein 1), is a highly-glycosylated protein that plays a role in endocytosis, modulates cellular events related to β-amyloid precursor protein metabolism, mediates kinase-dependent intracellular signaling, and is involved in neuronal calcium signaling as well as neurotransmission (Mao et al., 2017). LRP1 has been linked to a causative role in breast cancer susceptibility based on ethnic origins (Beneš et al., 2003). A third highly flux-responsive protein involved in signal transduction (albeit not a receptor *per se*) is **NCSTN** (nicastrin). Nicastin is an essential subunit of the γ-secretase complex that catalyzes intramembrane cleavage of receptors involved in Notch signaling in a glycosylation- and sialic acid-dependent manner (Yu et al., 2000; Moniruzzaman et al., 2018). Nicastrin modulates the epithelial to mesenchymal transition and tumorigenicity in breast cancer cells (Lombardo et al., 2012, 2014). Finally, we identified two plexins (**PLXNB2**, plexin B2 and **PLXND1**, plexin D1). Plexins are proteins that function as receptors for semaphorin signaling proteins that play important roles in neuronal development (e.g., axonal guidance) (Janssen et al., 2012). In cancer plexins can be either oncogenic or tumor suppressors; for example, plexins A1-4 are tumor suppressors while plexin-B2 is tumor promoting (Ramesh et al., 2018) and plexin D1 has been associated with tumor vasculature (Roodink et al., 2009).

#### Cell Adhesion (Figure 4B)

Sialic acid is strongly associated with cell adhesion. Indeed, intracellular levels of sialic acid previously have been linked to neuronal cell adhesion molecule (NCAM) sialylation (Bork et al., 2005), a protein similar to L1 cell adhesion molecule (**L1CAM**) identified in the current study. Consequently, we found it unsurprising that flux-based changes to sialylation affected molecules in this category. In health, L1CAM is an axonal glycoprotein involved in the dynamics of cell adhesion and in the generation of transmembrane signals at tyrosine kinase receptors. In cancer, it is an established biomarker for triple negative, advanced cancers with poor prognosis (Doberstein et al., 2014; Altevogt et al., 2016) specifically due to changes in sialylation linked to metastasis (Hoja-Łukowicz et al., 2013). A second cell adhesion protein we identified was FAT atypical cadherin 1 (**FAT1)**, a highly glycosylated cadherin-like protein that plays a role in cell migration, lamellipodia dynamics, cell polarity, and cell-cell adhesion (Katoh, 2012; Zhang et al., 2016). FAT1 repression in cancer occurs due to homozygous deletion or epigenetic silencing and is preferentially downregulated in invasive breast cancer (Katoh, 2012). Third, **TENM3** (Teneurin-3) is a single pass, richly glycosylated type II transmembrane protein that is one of four human Teneurins, a family involved in cell-cell adhesion and organization of neuronal synapses (Mosca, 2015; Jackson et al., 2018). Teneurins 2 and 4 have been linked to tumor differentiation and patient survival in ovarian cancer (Graumann et al., 2017) and Teneurin 3 is expressed at low to moderate levels in a subset of breast cancer patients (e.g., 4 of 12 reported in the Protein Atlas database, https://www.proteinatlas.org/ENSG00000218336-TENM3/pathology). Finally, **PTPRJ** (protein tyrosine phosphatase, receptor-type, J [*a.k.a*., receptor-type tyrosine-protein phosphatase eta]) negatively regulates PDGF, EGF, and VEGF signaling, and as such is a tumor suppressor gene (Smart et al., 2012). This protein's downstream targets play a role in cell-cell adhesion, cell-matrix adhesion, cell migration, cell adhesion, and barrier function of epithelial junctions during reassembly (Smart et al., 2012), thus positioning PTPRJ at the interface between signaling and cell adhesion (for the purposes of this discussion we arbitrarily included it in the cell adhesion category). Interestingly PTPRJ is one of 46 proteins we previously identified using 1,3,4-O-Bu_3_ManNAz (an azide-modified analog of 1,3,4-O-Bu_3_ManNAc) in the SW1990 pancreatic cancer line (Tian et al., 2015), indicating that this oncoprotein is responsive to flux through the sialic acid pathway across cancer and ManNAc analog types.

#### Protein Folding and Trafficking (Figure 4C)

In contrast to signal transduction and cell adhesion, the third category of highly flux-responsive proteins—molecular chaperones that assist in protein folding and other proteins involved in protein trafficking—was unexpected. For context, although these proteins—exemplified by calnexin and calreticulin—require glycopeptides as binding partners (Helenius and Aebi, 2001), relatively little is known about their own glycosylation (the only reports of glycosylation in online proteomic or genomic databases for two of the proteins identified in the current study came from an unrelated publication from our team; Hu et al., 2018). One of these proteins, **HYOU1** (hypoxia up-regulated protein 1), assists protein folding and secretion from the ER and has a pivotal role in cytoprotection during oxygen deprivation (Ikeda et al., 1997). This protein is highly expressed in the liver and pancreas, in macrophages found within aortic atherosclerotic plaques, and in breast cancer (Wang et al., 2015). A second member of this category, **FKBP10** (FKBP prolyl isomerase 10; Ishikawa et al., 2008) accelerates protein folding during synthesis. In cancer, aberrant epigenetic regulation of FKBP10 predicts poor clinical prognosis (Carmona et al., 2014). Third, **TXNDC11** (thioredoxin domain containing 11 protein) is another protein folding chaperone (Wang et al., 2005) linked to breast cancer through 2,522 mutations in the COSMIC database (as of November, 2018; https://cancer.sanger.ac.uk/cosmic/gene/analysis?ln=TXNDC11). Finally, **SORL1** (sortilin-related receptor) binds the receptor-associated protein and helps coordinate the cellular uptake, endosomal trafficking, and subsequent proteolytic processing of lipoproteins; in some cases such as the amyloid precursor protein, SORL1 impedes proteolytic processing (Rohe et al., 2008). (Note that we include SORL1 in this category because of its role in protein quality control and trafficking although unlike HYOU1, FKBP10, and TXNDC11 it is involved in protein recycling and degradation rather than biosynthesis). A “mutome” analysis identified SORL1 to be down-regulated in breast cancer (Hernández et al., 2007), suggesting that it may be a tumor suppressor.

An interesting aspect of the three molecular chaperones identified as being highly flux responsive (i.e., HYOU1, FKBP10, and TXNDC11; Figure 4C) was their fewer number of predicted sites of N-glycosylation compared to proteins related to signaling (Figure 4A) and adhesion (Figure 4B). Specifically all three of these proteins had fewer than 10 consensus sequons for N-glycans while the other proteins had from 21 to 49 sequons, depending on the protein (Figure 4D). Despite this large number of potential sites of N-glycosylation, only about half of these sites were predicted to be occupied using NetNGly (Blom et al., 2004) (from 13 to 31), with only a subset of this latter group identified in this study (from 3 to 12). To present this data another way, only 33 or 29% (for cell signaling and cell adhesion proteins, respectively) of possible acceptor sites were occupied with sialylated N-glycans (Figure 4D). By comparison, the molecular chaperones had a smaller number of possible consensus sequons (from 7 to 9) but sialoglycan occupancy was higher (at 50%), suggesting that sialylation is important for their activity.

### Mechanism of 1,3,4-O-Bu_3_ManNAc-Based Control of N-glycan Sialylation

#### Transcript Profiling of SAMG Genes

**Figure 5:**
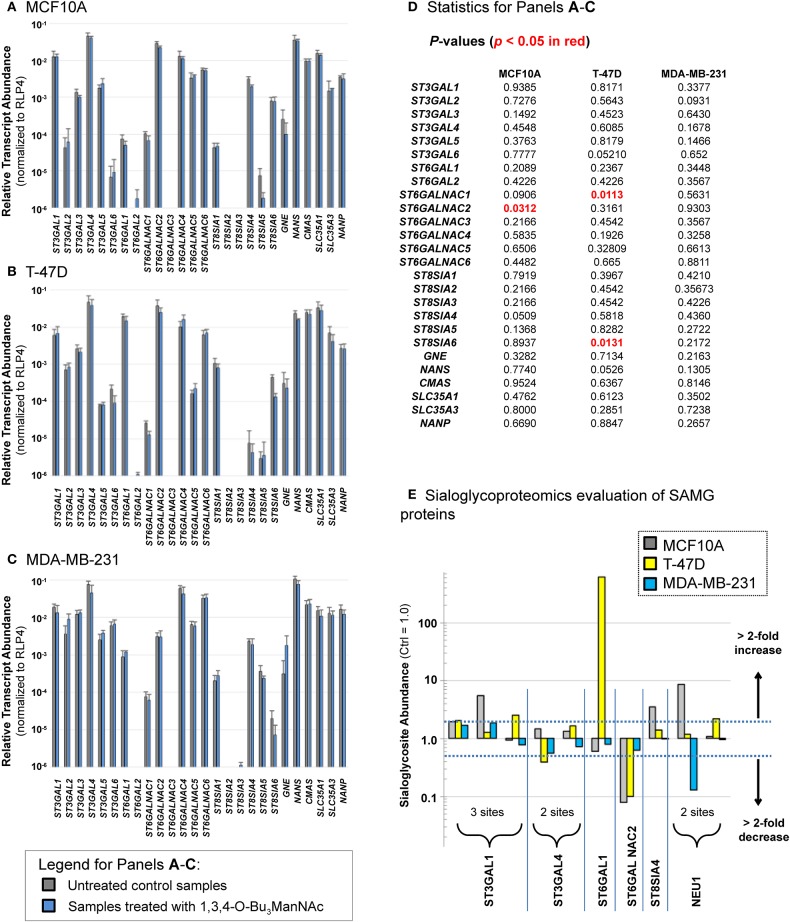
Transcript levels **(A–D)** and sialylation changes **(E)** observed for SAMG genes. Transcript levels of the SAMG genes were compared in the three breast lines: **(A)** MCF10A, **(B)** T-47D, and **(C)** MDA-MB-231 with and without incubation with 100 μM 1,3,4-O-Bu_3_ManNAc with *p-*values given in panel **(D)** with any statistically significant changes (*p* < 0.05) highlight in red font. **(E)** Sialoglycosites in SAMG proteins with > 2-fold changes in sialylation in at least one of the three cell lines are shown (the exact sites of these sialoglycosites are provided in Supplemental File S5). Transcript analyses were performed in triplicate. Student's *t*-tests were conducted to assess for significance for gene expression between control and 1,3,4-O-Bu_3_ManNAc treated samples.

The down-regulated sialoglycosites identified in Figure 4 were unexpected because, when flux through a metabolic pathway increases, product formation logically should increase in tandem. Our previous study with SW1990 cells was consistent with this premise, failing to identify any sialoglycosites with decreased abundance upon 1,3,4-O-Bu_3_ManNAc treatment (Almaraz et al., 2012b). A possible explanation for the apparently anomalous results in the current study was that butyrate released from 1,3,4-O-Bu_3_ManNAc epigenetically modulated gene expression through changes to histone acetylation (Sampathkumar et al., 2006) and affected transcription of SAMG genes in the breast lines differently than in SW1990 pancreatic cancer cells. This mechanism is plausible because 1,3,4-O-Bu_3_ManNAc can simultaneously up- and down-regulate transcription (Elmouelhi et al., 2009). Experimentally, profiling of SAMG genes revealed differences in transcript levels of SAMG genes between the cell lines (Figure 5, data for the MCF10A, T-47D, and MDA-MB-231 lines are shown in Panels **A**, **B**, and **C** respectively, with statistical analyses in Panel **D**). Such differences were consistent with basal differences in sialylation observed in each cell line (e.g., as shown in the clustering analysis of untreated cells in Figure 3). More germane to the flux-based glycoengineering evaluated in this paper, however, was that the transcript levels for SAMG genes changed relatively little (if any) upon analog supplementation as evidenced by the side-by-side comparison of each gene with and without 1,3,4-O-Bu_3_ManNAc treatment where only four statistically significant changes were observed (Figure 5D).

Having ruled out overt effects on transcription, we reasoned that glycosite sialylation could be affected by the availability of CMP-Neu5Ac in the Golgi, which can selectively activate subsets of cell's repertoire of STs toward individual glycosites (presumably each glycosite:ST interaction has an individualized K_M_ value) (Legaigneur et al., 2001; Gupta et al., 2017). Alternatively, the activity of the SAMG gene products themselves could be altered by flux-driven sialylation; increased esterase activity upon enhanced sialylation in 1,3,4-O-Bu_3_ManNAc-treated cells provides precedent for the flux-based control of a sialylated protein's activity (Mathew et al., 2017) along with evidence that sialylation of STs can impact their activity (Breen, 2002). To test if flux-based sialylation was relevant to STs and other SAMG proteins evaluated in the current study, we analyzed our dataset and found 10 sialoglycosites in six SAMG proteins, five of which were STs (Figure 5E). Although many of the changes were modest, 12 (of the 30 possible changes) exceeded a 2-fold change and were plausibly biologically significant.

Three of these proteins were particularly noteworthy. First, by far the largest change (of 619-fold) was for ST6GAL1 in the T-47D line; this glycosite is essentially unsialylated in untreated cells (i.e., sialylation must be <0.16% under basal conditions to observe a 619-fold increase). If increased sialylation at this site is activating, this change is consistent with the oncogenic role of this ST as described in several publications from the Bellis group (Zhuo and Bellis, 2011; Schultz et al., 2012, 2016 and corroborated by others; Meng et al., 2013). Conversely, sialylation of ST6GALNAC2 was strongly down-regulated upon 1,3,4-O-Bu_3_ManNAc treatment in both the MCF10A and T-47D lines but barely affected in the MDA-MB-231 line. The evaluation of global sialoglycosite abundance (as presented next, below) suggests that sialylation of this enzyme may be inactivating. Specifically, if sialylation is inactivating, the higher sialylation of this enzyme in MDA-MB-231 cells could explain the overall lower siaylation in this line. Finally, the recycling enzyme neuramidase 1 (NEU1) experienced diametrically opposed sialylation at one glycosite with strong up-regulation in the near-normal MCF10A line and strong down-regulation in the advanced MDA-MB-231 line. In this case if sialylation is inactivating, the less sialylated form of NEU1 in MDA-MB-231 cells would have enhanced activity, consistent with the lower levels of sialylated N-glycans in this line.

#### “Global” Analysis of Sialoglycopeptides

**Figure 6:**
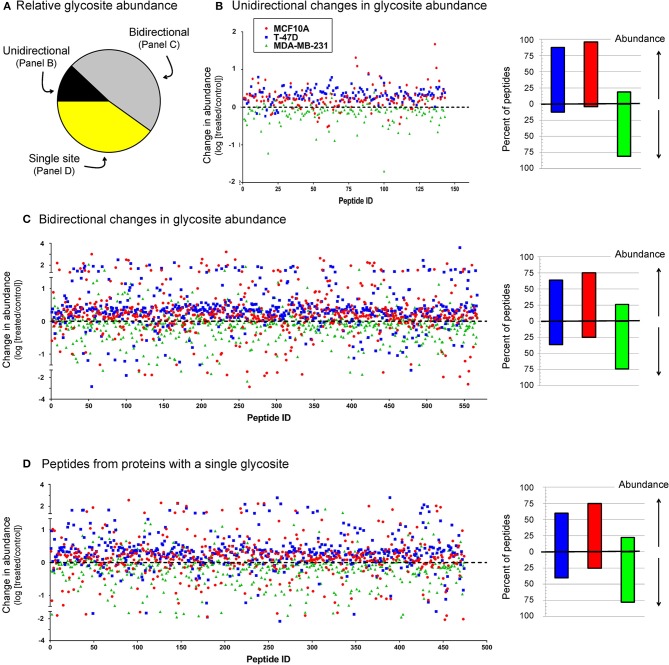
Relative sialoglycosite abundance **(A)** and scatter plots of sialoglycosites categorized on unidirectional **(B)**, bidirectional **(C)**, or singly-modified **(D)** glycoproteins. This data illustrates how the up- or down-regulation of sialylation at any specific glycosite is not (necessarily) related to the host protein because there is no clear trend between these categories (i.e., each category is qualitatively similar) but is more linked to the host cell line (i.e., the green dots from the MDA-MB-231 samples disproportionately cluster below 0 on the y-axis, indicating down-regulation).

As discussed above, many sialoglycosites experienced increased sialylation upon 1,3,4-O-Bu_3_ManNAc supplementation consistent with the increased intracellular production of sialic acid in the treated cells treated (Figure 2). By contrast, the decreased abundance of other sialoglycosites was unexpected because of the intuitive expectation that elevated levels of intracellular sialic acid would enhance sialoglycoconjugate formation. The surprising decreases in sialylation could be due to increased sensitivity of mass spectrometry that now allows identification of low abundance outliers with atypical sialylation or could result from the different cell types now analyzed (i.e., breast compared to pancreatic cancer cells) compared to our earlier study with pancreatic SW1990 cells (Almaraz et al., 2012b). Alternatively, the currently observed decreases could be artifacts of the experimental process; we reasoned that if technical issues were responsible similar trends would be observed across all cell lines. Accordingly, we plotted all sialoglycosite-specific changes observed upon 1,3,4-O-Bu_3_ManNAc treatment in the three lines (Figure 6). We first compared the number of proteins that generated single sialoglycopeptides with those that produce two or more sialoglycosites; the latter proteins were further categorized based on whether the sialoglycosites experienced uni- or bi-directional changes in abundance (Figure 6A). In subsequent panels, we provide dot representations showing the fold-change of each glycosite to compare responses in each cell line for each of these three categories with data for proteins with two or more sialoglycosites with unidirectional changes is given in Figure 6B; proteins that experienced bidirectional changes are shown in Figure 6C; and proteins that generated a single glycosite are shown in Figure 6D. In all cases, the majority of sialoglycosites identified in the MCF10A and T-47D lines (red and blue dots) clustered above 0 on the y-axis indicating increased abundance in 1,3,4-O-Bu_3_ManNAc-treated cells while glycosites from the MDA-MB-231 line (green dots) were disproportionately skewed below 0, indicating reduced sialylation.

**Figure 7:**
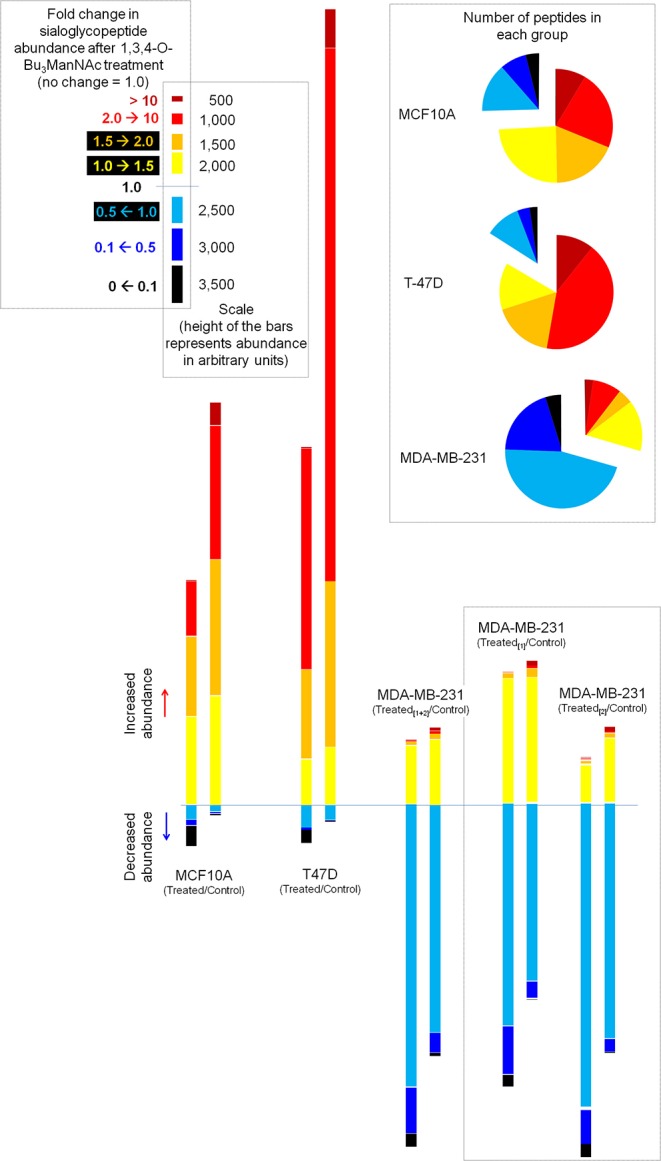
Aggregate levels of glycosites illustrating the overall changes in sialylation. The pie charts (inset) summarize the number of sialoglycosites in each category, essentially depicting the data from Figure 6 in another format. The bar graphs aggregate the sialoglycopeptides identified in each category by first normalizing for the abundance of the peptide and then scaling each peptide based on the degree of change in sialylation (i.e., in the 2.0→10 category, a sialopeptide that increased by 8-fold upon treatment with 1,3,4-O-Bu_3_ManNAc would be weighted 4-fold more than a sialoglycopeptide that increased only by 2-fold). Duplicate evaluation of the MDA-MB-231 line showed that this methodology showed measurable quantitative differences but the qualitative differences between this line and the other two remained very discernible in either replicate.

We next normalized sialoglycopeptides based on abundance and then scaled and summed these values to represent the aggregate abundance of up- and down-regulated sialopeptides in each line (Figure 7). The results reinforced that both the MCF10A and T-47D cell lines responded as expected with the preponderance of glycopeptides experiencing increased sialylation upon 1,3,4-O-Bu_3_ManNAc treatment. In addition, the aggregate increase in sialylation was more pronounced in the T-47D line compared to the MCF10A line, consistent with the increased amount of intracellular sialic acid produced in T-47D cells (Figure 2). Similarly, the hierarchal clustering analysis showed that more peptides with decreased sialylation occurred in the near-normal MCF10A line (e.g., in the topmost cluster, Figure 3B). By contrast to the T-47D cancer and the near-normal MCF10A lines, the MDA-MB-231 cells did not show a trend toward increased sialylation in the clustering analysis (Figure 3B) even though 1,3,4-O-Bu_3_ManNAc did support a clearly-measurable increase in intracellular sialic acid in this advanced cancer line (Figure 2B). A possible explanation for this unexpected result was that the glycoproteins experiencing decreased sialylation upon 1,3,4-O-Bu_3_ManNAc treatment were disproportionately low in abundance and did not reflect global sialylation. The data shown in Figure 7, however, discounts this possibility because the scaled representation of aggregate sialoglyconjugate abundance showed a clear decrease in global sialylation in the MDA-MB-231 cells that was reproduced in two independent experiments.

## Discussion

The sialoglycosite analyses of 1,3,4-O-Bu_3_ManNAc-treated human breast cell lines highlights—and further reinforces—a coalescing consensus that metabolic flux can influence sialylation in biologically-meaningful and disease-relevant ways. Recently, flux-based modulation of sialylation has been shown to be important in several contexts. For example, media supplementation with ManNAc (Yorke, 2013) or our analog (i.e., 1,3,4-O-Bu_3_ManNAc) improves glycan product quality for therapeutic glycoproteins (Wang et al., 2019) (a similar approach used sialuria-type mutations to GNE to increase sialylation; Bork et al., 2007). In this report we focus on a second context, which is flux-driven sialylation in cancer. In the past we have used sialoglycosite analysis to characterize 1,3,4-O-Bu_3_ManNAc-treated SW1990 pancreatic cancer cells (Almaraz et al., 2012b), and in follow up studies, showed that these changes altered oncogenic signal transduction and sensitivity to drugs (Mathew et al., 2015, 2017).

In the current study we used a metabolic glycoengineering strategy to uncover numerous flux-based perturbations to sialylation exemplified by the 1410 sialylated glycopeptides identified in common between MCF10A, T-47D, and MDA-MB-231 cells. Overall, these changes affected proteins of almost all classes and activities. The categories of biological activity, however, became tightly focused in the small subset of 13 highly flux-responsive proteins that exhibited two (or more) changes in sialylation of at least 10-fold (Figure 4). Two of these categories (cell signaling and cell adhesion) were expected because of literature precedent implicating sialic acid in these processes in cancer. The third category (protein folding chaperones) was not expected because very little information is available on the glycosylation, let alone the sialylation, of these proteins. Accordingly, the discovery that molecular chaperone proteins are highly responsive to flux-based sialylation opens a new frontier for exploring (and ultimately controlling) the function of these proteins.

Another aspect of the highly flux-responsive set of proteins shown in Figure 4 is that they all are linked to cancer and in most cases, specifically to breast cancer. Further, several are either tumor suppressors or, depending on context, proto-oncogenes. The controlling factors that determine whether tumor suppressor or pro-oncogenic activity dominates the function of these proteins largely remains obscure; our current study opens the intriguing possibility that sialylation is a switch that turns either behavior on or off. Although speculative, exquisite control of sialylation may provide cells with the ability to tune the activity of “context dependent” tumor suppressor or proto-oncogenic proteins (Katoh, 2012; Li and Reynolds, 2012) and thus provide a cancer-driving stimuli or tumor inhibition depending on glycosylation status. The ability of cells to tune biological activity (such as tumor suppression vs. cancer progression) is supported by the high resolution characterization (as shown in Figure 4 for selected proteins and Figure 6 for all sialoglycosites) that shows that metabolic flux has a remarkable ability to selectively increase, decrease, or avoid perturbing sialylation. We emphasize that this selectivity occurs at individual sialoglycosite level (as can be seen by the comparisons made in Figure 6 where no discernible difference is evident for proteins with single sialoglycosites or multi-sialoglycosite proteins with either uni- or bi-directional changes). Global patterns of sialylation are nonetheless heavily influenced by the host cell line, which is evident in Figure 7 where 1,3,4-O-Bu_3_ManNAc treatment induces a modest enhancement of sialylation in the near-normal MCF10A line, a strong increase in the cancerous T-47D line, and the unexpected global decrease in the advanced triple negative MDA-MB-231 line.

Although decreased abundance was observed at dozens of sialoglycosites in the near-normal MCF10A and T-47D lines, the overall response of these lines nevertheless fit the canonical understanding of the role of sialic acid in oncogenesis. Specifically the minor increase in intracellular metabolites as detailed previously (Saeui et al., 2018) and confirmed in the present study in the near-normal MCF10A line translated into a substantial increase in sialoglycoconjugates at the global level (as shown in Figures 6, 7). Considering the long-established links between sialylation and oncogenesis, these data suggest that flux driven sialylation could be an early triggering factor in the development of cancer. The cancerous T-47D line has an increased ability to produce intracellular sialometabolites (Figure 2), which in turn increases sialoglycoconjugate production substantially beyond levels seen in the near-normal MCFA10A line, consistent with the many known roles of sialic acid in cancer progression. Indeed, we speculate that the copious production of this sugar in the T-47D line could help drive these cancer cells to more advanced and malignant forms of this disease. The surprising aspect of this study comes into play with the advanced MDA-MB-231 line where there was a substantial global decrease in sialylated glycosites (Figures 6, 7).

To ensure that the decrease in sialylation observed in the MDA-MB-231 line was a legitimate metabolic flux-based effect we repeated glycosite analysis in this line with quantitatively similar results (see Figure 7). We also ruled out that 1,3,4-O-Bu_3_ManNAc treatment reduced transcript levels of SAMG genes (Figure 5), suggesting that cells maintained the ability to produce the biosynthetic machinery (i.e., the various enzymes and transporters shown in Figure 1) required to synthesize sialylated glycans. Instead, an interesting hypothesis is that reduced sialoglycan levels may be a consequence of the sialylation status of the SAMG proteins themselves (Figure 5E). Finally, the MDA-MB-231 line has been analyzed by the Affymetrix Human Genome U133 2.0 Plus chip by using the protocols and facilities available through the Johns Hopkins Cancer Center Microarray Core [the resulting data were deposited in NCBI's Gene ExpressionOmnibus database (Edgar et al., 2002) and are accessible through GEO series accession number GSE11407 [http://www.ncbi.nlm.nih.gov/geo/query/acc.cgi?acc]]. This data showed minimal global perturbation of transcript levels in 1,3,4-O-Bu_3_ManNAc treated MDA-MB-231 lines (Elmouelhi et al., 2009), further supporting the idea that that differences we observed were due to flux-based changes that affected post-translational sialylation.

We close by speculating on why advanced cancer cells—exemplified by MDA-MB-231 in this study—may benefit by down-regulating sialylated N-glycans. We hypothesize that sialic acid in cancer is an example of a “Goldilocks” effect where levels have to be “just right” (vitamins, which are critical to maintain health but often become toxic at higher doses, exemplify this paradigm in a different health context; Diab and Krebs, 2018). In particular, although sialylation drives multiple aspects of oncogenesis, too large of an increase similarly may be detrimental. This idea is consistent with descriptions of only “slightly increased” levels of sialic acid in some types of cancer (Sillanaukee et al., 1999) and feedback mechanisms that carefully titer metabolic flux into the sialic acid biosynthetic pathway (Kornfeld et al., 1964; Keppler et al., 1999).

Once cancer cells achieve an advanced stage, they may no longer require sialic acid as an oncogenic driving force. Instead, sialylation can become a liability. One example of this phenomenon is provided by the SW1990 pancreatic cancer cell line where oncogenic EGFR signaling is dampened by 1,3,4-O-Bu_3_ManNAc-driven sialylation (Mathew et al., 2016). Increased flux-driven sialylation also sensitizes this drug-resistant line to tyrosine kinase inhibitors (e.g., erlotinib and gefitinib; Mathew et al., 2015). Related to the current study, the MDA-MB-231 cancer line is of breast origin but obtained from a distal metastatic site; this may help explain why this line down-regulates sialylation. Specifically, because sialic acid inhibits cell extravasation through endothelium (Cross et al., 2003; Sakarya et al., 2004; French et al., 2017), MDA-MB-231 cells evolved mechanisms to reduce sialylation to facilitate exit from the vascular to form secondary tumors at distal sites during metastasis. Interestingly, sialidases (e.g., NEU1, identified in this study to undergo flux-driven changes to sialylation in MDA-MB-231 cells in this study, Figure 5E) play a major role in cell migration across endothelia (Cross et al., 2003, 2012; Sakarya et al., 2004).

In conclusion, the glycoengineering approach taken in this report has uncovered novel, and in some cases completely unexpected, roles for flux-based sialylation in breast cancer. One striking result was the down-regulation of dozens of sialoglycosites upon treatment with the metabolite precursor (1,3,4-O-Bu_3_ManNAc) that feeds the sialic acid biosynthetic pathway. Another intriguing result was that the subset of 13 proteins that were highly flux-responsive are all linked to cancer, in many cases specifically to breast cancer, which validates our metabolic glycoengineering approach as an appropriate strategy to uncover insights into how sialylation affects this disease. Finally, although the function and activity of a few of the highly flux-responsive proteins have already been linked to glycosylation in general and sialylation more specifically (e.g., IGF2F and L1CAM), virtually nothing is currently known about the role of glycans for most of the other proteins, in particular for the protein folding chaperones (i.e., HYOU1, FKBP10, and TXNDC11).

Based on these considerations, this report establishes a set of sialoglycoproteins as novel targets for investigation into how glycosylation impacts their biological activity in both health and malignant disease. Consequently, this work augments growing efforts to translate metabolic glycoengineering into clinical healthcare (Agatemor et al., 2019). Although entirely speculative at this point, one way our 1,3,4-O-Bu_3_ManNAc-based approach can be envisioned to be deployed clinically is through emerging *in vitro* functional assays of a patient's living cancer cells (Kodack et al., 2017). More specifically, cancer cells obtained from a biopsy can be incubated with 1,3,4-O-Bu_3_ManNAc (or similar agent) to reveal otherwise hidden biochemical features that—if the trends in sialylation observed for the three cell lines evaluated in this study hold—can be used to assess the stage of cancer progression and use this information for personalized patient care.

## Data Availability Statement

All datasets generated for this study are included in the article/Supplementary Material.

## Author Contributions

CS and KY provided project design and management. KC provided mass spectrometry data acquisition. AN and MG conducted SAMG transcript profiling experiments. CS, VD, SS, MP, MA, AC, EC, MB, and RA conducted all other experiments. KY, HZ, and KM were responsible for funding acquisition. CS, KC, KY, AN, KM, and HZ participated in writing and editing of the manuscript.

### Conflict of Interest

The authors declare that the research was conducted in the absence of any commercial or financial relationships that could be construed as a potential conflict of interest.

## Supplementary Material

Supplemental File S1 Characterization of 1,3,4-O-Bu_3_ManNAc.

Supplemental File S2 Primers for SAMG gene transcript quantification. Validated primer sequences for all 20 STs is provided here.

Supplemental File S3 Sialoglycosite analysis. Raw mass spectrometry data from SPEG experiments for protein abundance and identified peptide sequences containing sialoglycosites is provided.

Supplemental File S4 GO analysis. Overview of PANTHER (http://www.pantherdb.org/) generated GO results of SPEG analyzed samples.

Supplemental File S5 Glycosite annotation. This file provides precise identification of glycosites highlighted in Figures 4, 5.

## Abbreviations

FAT1: FAT atypical cadherin 1
FKBP10: FKBP prolyl isomerase 10
GNE: UDP-GlcNAc 2-epimerase/ManNAc kinase
GO: gene ontology
HYOU1: hypoxia up-regulated protein 1
IGF2R: insulin-like growth factor 2 receptor
L1CAM: L1 cell adhesion molecule
LRP1: low-density lipoprotein receptor-related protein 1
NANP: Neu5Ac 9-phosphate phosphatase
NANS: Neu5Ac 9-phosphate synthase
NCSTN: nicastrin
NEU1: neuramidase 1
PTPRJ: protein tyrosine phosphatase receptor-type J
SAMG: sialic acid metabolism and glycosylation
SLC35A1: cytidine 5′-monophosphate (CMP)-sialic acid transporter
SLC35A3: UDP-N-acetylglucosamine transporter
SORL1: sortilin-related receptor
SPEG: solid phase extraction of glycopeptides
ST: sialyltransferase
SFCA: short chain fatty acid
TENM3: teneurin-3
TXNDC11: thioredoxin domain containing 11 protein.
