# Supplementary material for: Cell Line-, Protein-, and Sialoglycosite-Specific Control of Flux-Based Sialylation in Human Breast Cells: Implications for Cancer Progression
Source: Front Chem. 2020 Feb 5;8:13. doi: 10.3389/fchem.2020.00013 (PMC7013041; doi:10.3389/fchem.2020.00013)
Supplement: Supplemental File S1 — Characterization of 1,3,4-O-Bu3ManNAc. [file Data_Sheet_1.pdf]

Supplemental File S1 *for*

Cell Line-, Protein-, and Sialoglycosite-Specific Control of Flux-Based Sialylation in Human Breast Cells: Implications for Cancer Progression

doi: 10.3389/fchem.2020.00013

Characterization of 2-acetamido-1,3,4-tri-*O*-butanoyl-2-deoxy-α-D-mannopyraose (**1,3,4-O-Bu$_3$ManNAc**).

Semi-solid. ^{1}H-NMR (400 MHz, $CDCl_3$): δ 6.11 (d, 1H, J = 9.6 Hz, NH), 6.07 (d, 1H, J = 2.0 Hz, H-1), 5.43 (dd, 1H, J = 4.8 & 10.4 Hz, H-3), 5.19 (t, 1H, J = 10.4 Hz, H-4), 4.68 (m, 1H, H-2), 3.82 (m, 1H, H-5), 3.75 (m, 1H, H-6a), 3.60 (m, 1H, H-6b), 2.75 (m, 1H, C6-OH), 2.50-2.15 (m, 6H, 3 x CH_2), 2.08 (s, 3H, NHAc), 1.80-1.50 (m, 6H, 3 x CH_2), 1.08-0.80 (m, 9H, 3 x CH_3); ^{13}C-NMR (100 MHz, $CDCl_3$): δ173.8, 172.6, 171.0, 170.2 (NH$\underline{C}$O), 91.8 (C-1), 72.5, 68.4, 65.5, 60.8 (C-6), 49.4 (C-2), 36.0, 36.0, 35.9, 23.2, 18.4, 18.2, 18.1, 13.6, 13.6, 13.6. Calc'd for $C_{20}H_{33}NO_9Na$ ($[M^+Na]^+$): 454.2053, found: 454.2033.
