# Supplementary material for: Cell Line-, Protein-, and Sialoglycosite-Specific Control of Flux-Based Sialylation in Human Breast Cells: Implications for Cancer Progression
Source: Front Chem. 2020 Feb 5;8:13. doi: 10.3389/fchem.2020.00013 (PMC7013041; doi:10.3389/fchem.2020.00013)
Supplement: Supplemental File S2 — Primers for SAMG gene transcript quantification. Validated primer sequences for all 20 STs is provided here. [file Data_Sheet_2.pdf]

Supplemental File S2 *for*

Cell Line-, Protein-, and Sialoglycosite-Specific Control of Flux-Based Sialylation in Human Breast Cells: Implications for Cancer Progression

doi: 10.3389/fchem.2020.00013

Gene symbol	Gene ID	Forward Primer (5' to 3')	Reverse Primer (5' to 3')	Accession #
ST3GAL1	6482	ATTGAGCCTCCACCTCTGG	CACGATGGGTAGCAGGAACT	NM_003033, NM_173344
ST3GAL2	6483	TGGTTTGACAGCCACTTTGA	CGGTGGAAGATCCATGTTCT	NM_006927
ST3GAL3	6487	ACACTGCGCATCACCTACC	GAGAGAATCGCGCTCGTACT	NM_001270459, NM_001270460, NM_001270461, NM_001270462, NM_001270463, NM_001270464, NM_001270465, NM_001270466, NM_001350619, NM_001350620, NM_001350621, NM_006279, NM_174963, NM_174964, NM_174965, NM_174966, NM_174967, NM_174968, NM_174969, NM_174970, NM_174971
ST3GAL4	6484	CGTCCTGGTAGCTTTCAAGG	GCACCCGCTTCTTATCACTC	NM_006278, NM_001254757,NM_001254758, NM_001254759
ST3GAL5	8869	CAACGGAAACCAAGTTCCTCT	CACGATCAATGCCTCCACT	NM_003896, NM_001042437
ST3GAL6	10402	CCTAAACACCCAACAACAGGA	CCAGCTAGGTGAACTTCGTGA	NM_006100, NM_001271142, NM_001271145, NM_001271146, NM_001271147,NM_001271148
ST6GAL1	6480	TATCTGCCCAAGGAGAGCAT	GACGACACAACAGCACACCT	NM_003032, NM_173216, NM_173217
ST6GAL2	84620	CTGTACCGGCTCTGGAAGG	ATCCTTCATCGCCTTCTGC	NM_032528, NM_001142351,NM_001142352
ST6GALNAC1	55808	AACAATGCCCTCAACACACA	GCCTCCTTTCCTCTGTCTCC	NM_018414, NM_001289107
ST6GALNAC2	10610	GATGTGGGCACCAAGACTTC	CCAGTAGGAGACGAGGGAGTT	NM_006456
ST6GALNAC3	256435	TGGTTGTGCGTCTTGTAAATG	CCACTTTGTACCAGGTTGTCC	NM_152996, NM_001160011
ST6GALNAC4	27090	CCCTCAGTGCCTTACCACTACT	CTGCTCGTGTGCCAGGT	NM_014403, NM_175039, NM_175040
ST6GALNAC5	81849	CAACGGAAACCAAGTTCCTCT	TGCCTCCACTGAGATCTTTCA	NM_030965
ST6GALNAC6	30815	GAGCGGGCTGAGTGTACAA	GGTCTTGTTGCCCACATCA	NM_013443, NM_001286999,NM_001287000, NM_001287001, NM_001287002,NM_001287003
ST8SIA1	6489	GCTGTACTGGCGTGGAAGTT	AGAGGACCACGACACAGAGG	NM_003034, NM_001304450
ST8SIA2	8128	CTCAATGGCAGCATCCTGT	CTCGTTGACCCACTCAACAC	NM_006011
ST8SIA3	51046	CAGCAACTGTGACCAGGACA	GCCAAGCCAGTTGGACTTT	NM_015879
ST8SIA4	7903	GAGGAGAGAAGCACGTGGAG	AGGCAGTTCGCACTTTCAGT	NM_005668, NM_175052
ST8SIA5	29906	GGTTCCACAAGCTGGAGAAG	ACGCGTTCTCGTACACCTG	NM_013305, NM_001307986,NM_001307987
ST8SIA6	338596	CGCTCGAAGAGTCTAAAGCAA	AAGGGCCAGATCTTTCAGGT	NM_001004470
GNE	10020	CTGGAACAGCTTTGGGTCTT	GATCACAAGGGAGGGATTCA	NM_005476, NM_001128227,NM_001190383, NM_001190384, NM_001190388
NANS	54187	TTGGAGAGGCCATACACCTC	TCCAGATGTCGTTTGTGCTC	NM_018946
CMAS	55907	CCTGGCAGCCCTAATTCTG	CGCCAGGTGCTTAATGTTCT	NM_018686
NANP	140838	CAGACAGACCCAGAGGGAGA	CACCTACAACAACAGCGTCAA	NM_152667
SLC35A1	10559	TGCCATCGTTAGTGTATGCTG	CTGCTGCATCCAGATTGCTA	NM_006416, NM_001168398
SLC35A3	23443	GGAACTTTCAGCTGGTTCTCA	CCAGCAAAGCCACTTGAAA	NM_012243, NM_001271684,NM_001271685
RPL4	6124	AGAAGGCTGCTGTTGGTGTT	TGGTTTCTTGGTAGCTGCTG	NM_000968
