# Supplementary material for: Cell Line-, Protein-, and Sialoglycosite-Specific Control of Flux-Based Sialylation in Human Breast Cells: Implications for Cancer Progression
Source: Front Chem. 2020 Feb 5;8:13. doi: 10.3389/fchem.2020.00013 (PMC7013041; doi:10.3389/fchem.2020.00013)
Supplement: Supplemental File S4 — GO analysis. Overview of PANTHER (http://www.pantherdb.org/) generated GO results of SPEG analyzed samples. [file Data_Sheet_4.pdf]

Supplemental File S4 *for*

Cell Line-, Protein-, and Sialoglycosite-Specific Control of Flux-Based Sialylation in Human Breast Cells: Implications for Cancer Progression

doi: 10.3389/fchem.2020.00013

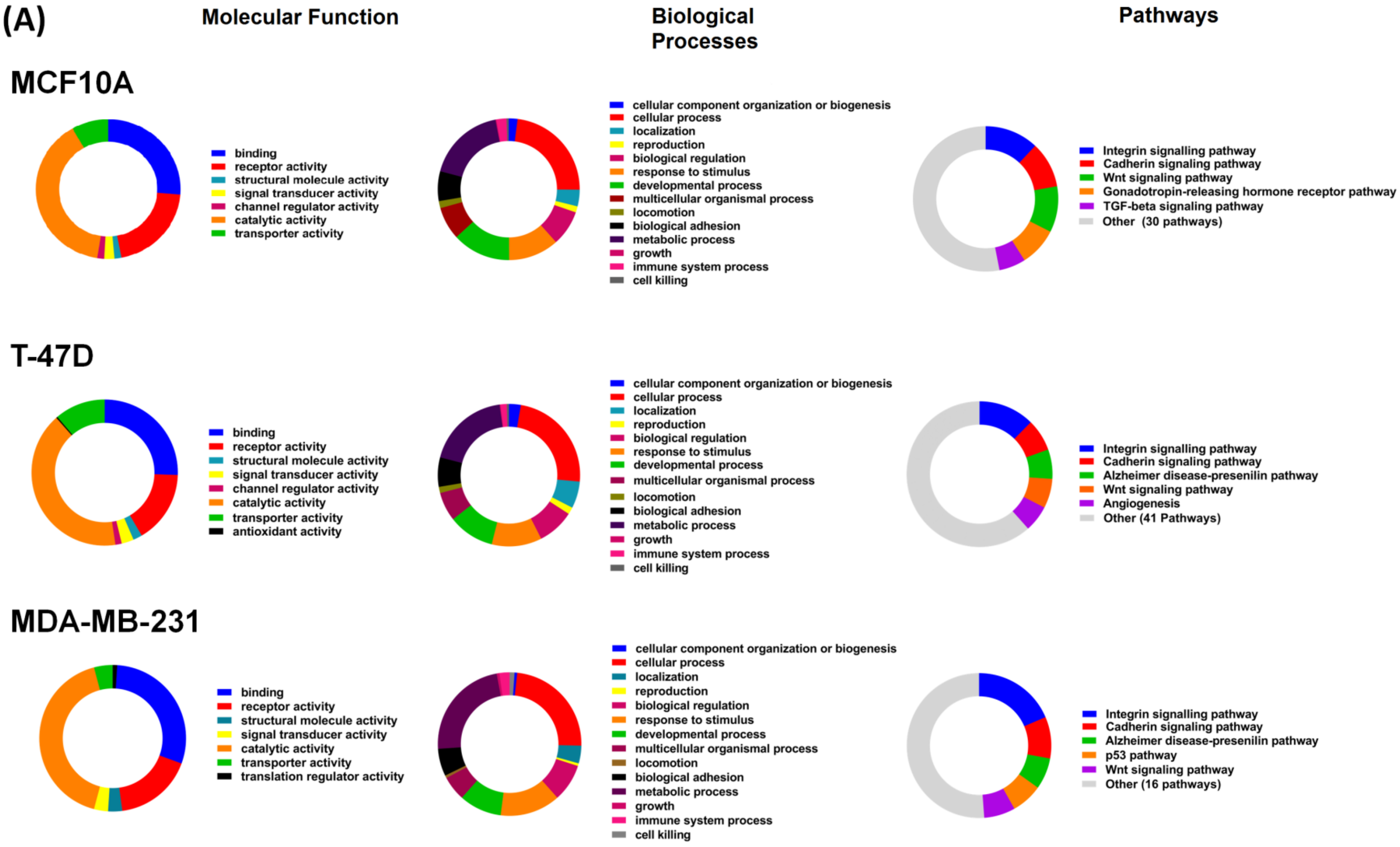

GO analysis. Overview of PANTHER (http://www. pantherdb.org/) generated GO results of SPEG analyzed samples.
