# Supplementary material for: Cell Line-, Protein-, and Sialoglycosite-Specific Control of Flux-Based Sialylation in Human Breast Cells: Implications for Cancer Progression
Source: Front Chem. 2020 Feb 5;8:13. doi: 10.3389/fchem.2020.00013 (PMC7013041; doi:10.3389/fchem.2020.00013)
Supplement: Supplemental File S5 — Glycosite annotation. This file provides precise identification of glycosites highlighted in Figures 4, 5. [file Data_Sheet_5.pdf]

Supplemental File S5 *for*

Cell Line-, Protein-, and Sialoglycosite-Specific Control of Flux-Based Sialylation in Human Breast Cells: Implications for Cancer Progression

doi: 10.3389/fchem.2020.00013

This document supplies supporting information for Figures 4 & 5

Glycosites shown in Figures 4 (panels A, B, & C) and Figure 5E are arbitrarily numbered (i.e., 1, 2, 3…. to “n”) and do not provide the exact site of the flux-responsive sialoglycan(s). This data is provided on the following pages, as follows:

Specifically, for Figure 4A, glycosite annotation data is provided:

- On Page 2 for IGF2R
- On Page 3 for LRP1
- On Page 4 for NCSTN
- On Page 5 for PLXNB2
- On Page 6 for PLXND1

For Figure 4B, glycosite annotation data is provided:

- On Page 7 for FAT1
- On Page 8 for L1CAM
- On Page 9 for TENM3
- On Page 10 for PTPRJ
- On Page 11 for SORL1

For Figure 4C, glycosite annotation data is provided:

- On Page 12 for HYOU1
- On Page 13 for FKBP10
- On Page 14 for TXNDC11
- On Page 15 for SORL1

For Figure 5E, glycosite annotation data is provided:

- On Page 16 for ST3GAL1
- On Page 17 for ST3GAL4
- On Page 18 for ST6GAL1
- On Page 19 for ST6GALNAC2
- On Page 20 for ST8SIA4
- On Page 21 for NEU1

IGF2R (cation-independent mannose-6-phosphate receptor)

Data ID[a] (Glycosite #)[b]	Peptide sequence	Ratio of sialoglycosite abundance in 1,3,4-O-Bu_3ManNAc-treated to control cells: MCF10A	T-47D	MDA-MB-231
**605[a] (1)[b]**	DAGVGFPEYQEED**NST**YNFR	0.03	2.00	144.81
**3950 (2)**	**NGS**SIVDLSPLIHR	1.57	2.42	1.25
**5307 (3)**	T**NIT**LVCK	1.43	1.12	1.21
**5156 (4)**	TGPVVEDSGSLLLEYV**NGS**ACTTSDGR	2.27	1.88	1.13
**5308 (5)**	T**NIT**LVCKPGDLESAPVLR	1.20	2.77	1.10
**4821 (6)**	SLLEF**NTT**VSCDQQGTNHR	1.74	2.60	0.96
**2126 (7)**	HF**NYT**SLIAFHCK	0.99	3.14	0.58
**5099 (8)**	TEGE**NCT**VFDSQAGFSFDLSPLTK	1.60	1.27	0.48
**3743 (9)**	MDGCTLTDEQLLYSF**NLS**SLSTSTFK	33.79	1.77	0.05

[a] The data ID number is provided in **Column A** of **Sheet 1** of **Supplemental File #3**

[b] The glycosite # represents the labeling scheme used in **Figure 4** of the main text

Amino acid sequence (*from* Uniprot; https://www.uniprot.org/uniprot/ P11717)

N-glycan consensus sequons in **red** are predicted by NetNGly to be occupied

N-glycan consensus sequons in **blue** are predicted by NetNGly to be NOT occupied

Sequences underlined using a dotted line represent peptide sequences identified as "glycosites"

```
        10         20         30         40         50
MGAAAGRSPH LGPAPARRPQ RSLLLLQLLL LVAAPGSTQA QAAPFPELCS
        60         70         80         90        100
YTWEAVDTKN NVLYKINICG SVDIVQCGPS SAVCMHDLKT RTYHSVGDSV
       110        120        130        140        150
LRSATRSLLE FNTTVSCDQQ GTNHRVQSSI AFLCGKTLGT PEFVTATECV   ← Glycosite 4821 (6) [@N112]
       160        170        180        190        200
HYFEWRTTAA CKKDIFKANK EVPCYVFDEE LRKHDLNPLI KLSGAYLVDD
       210        220        230        240        250
SDPDTSLFIN VCRDIDTLRD PGSQLRACPP GTAACLVRGH QAFDVGQPRD
       260        270        280        290        300
GLKLVRKDRL VLSYVREEAG KLDFCDGHSP AVTITFVCPS ERREGTIPKL
       310        320        330        340        350
TAKSNCRYEI EWITEYACHR DYLESKTCSL SGEQQDVSID LTPLAQSGGS
       360        370        380        390        400
SYISDGKEYL FYLNVCGETE IQFCNKKQAA VCQVKKSDTS QVKAAGRYHN
       410        420        430        440        450
QTLRYSDGDL TLIYFGGDEC SSGFQRMSVI NFECNKTAGN DGKGTPVFTG
       460        470        480        490        500
EVDCTYFFTW DTEYACVKEK EDLLCGATDG KKRYDLSALV RHAEPEQNWE
       510        520        530        540        550
AVDGSQTETE KKHFFINICH RVLQEGKARG CPEDAAVCAV DKNGSKNLGK
       560        570        580        590        600
FISSPMKEKG NIQLSYSDGD DCGHGKKIKT NITLVCKPGD LESAPVLRTS   ← Glycosite 5307 (3) [@N581]
                                                         ← Glycosite 5308 (5) [@N581]
       610        620        630        640        650
GEGGCFYEFE WHTAAACVLS KTEGENCTVF DSQAGFSFDL SPLTKKNGAY   ← Glycosite 5099 (8) [@N626]
       660        670        680        690        700
KVETKKYDFY INVCGPVSVS PCQPDSGACQ VAKSDEKTWN LGLSNAKLSY
       710        720        730        740        750
YDGMIQLNYR GGTPYNNERH TPRATLITFL CDRDAGVGFP EYQEEDNSTY   ← Glycosite 605 (1) [@N747]
       760        770        780        790        800
NFRWYTSYAC PEEPLECVVT DPSTLEQYDL SSLAKSEGGL GGNWYAMDNS
       810        820        830        840        850
GEHVTWRKYY INVCRPLNPV PGCNRYASAC QMKYEKDQGS FTEVVSISNL
       860        870        880        890        900
GMAKTGPVVE DSGSLLLEYV NGSACTTSDG RQTTYTTRIH LVCSRGRLNS   ← Glycosite 5156 (4) [@N871]
       910        920        930        940        950
HPIFSLNWEC VVSFLWNTEA ACPIQTTTDT DQACSIRDPN SGFVFNLNPL
       960        970        980        990       1000
NSSQGYNVSG IGKIFMFNVC GTMPVCGTIL GKPASGCEAE TQTEELKNWK
      1010       1020       1030       1040       1050
PARPVGIEKS LQLSTEGFIT LTYKGPLSAK GTADAFIVRF VCNDDVYSGP
      1060       1070       1080       1090       1100
LKFLHQDIDS GQGIRNTYFE FETALACVPS PVDCQVTDLA GNEYDLTGLS
      1110       1120       1130       1140       1150
TVRKPWTAVD TSVDGRKRTF YLSVCNPLPY IPGCQGSAVG SCLVSEGNSW
      1160       1170       1180       1190       1200
NLGVVQMSPQ AAANGSLSIM YVNGDKCGNQ RFSTRITFEC AQISGSPAFQ
      1210       1220       1230       1240       1250
LQDGCEYVFI WRTVEACPVV RVEGDNCEVK DPRHGNLYDL KPLGLNDTIV
      1260       1270       1280       1290       1300
SAGEYTYYFR VCGKLSSDVC PTSDKSKVVS SCQEKREPQG FHKVAGLLTQ
      1310       1320       1330       1340       1350
KLTYENGLLK MNFTGGDTCH KVYQRSTAIF FYCDRGTQRP VFLKETSDCS
      1360       1370       1380       1390       1400
YLFEWRTQYA CPPFDLTECS FKDGAGNSFD LSSLSRYSDN WEAITGTGDP
      1410       1420       1430       1440       1450
EHYLINVCKS LAPQAGTEPC PPEAAACLLG GSKPVNLGRV RDGPQWRDGI
      1460       1470       1480       1490       1500
IVLKYVDGDL CPDGIRKKST TIRFTCSESQ VNSRPMFISA VEDCEYTFAW
      1510       1520       1530       1540       1550
PTATACPMKS NEHDDCQVTN PSTGHLFDLS SLSGRAGFTA AYSEKGLVYM
      1560       1570       1580       1590       1600
SICGENENCP PGVGACFGQT RISVGKANKR LRYVDQVLQL VYKDGSPCPS
      1610       1620       1630       1640       1650
KSGLSYKSVI SFVCRPEARP TNRPMLISLD KQTCTLFFSW HTPLACEQAT
      1660       1670       1680       1690       1700
ECSVRNGSSI VDLSPLIHRT GGYEAYDESE DDASDTNPDF YINICQPLNP   ← Glycosite 3950 (2) [@N1656]
      1710       1720       1730       1740       1750
MHGVPCPAGA AVCKVPIDGP PIDIGRVAGP PILNPIANEI YLNFESSTPC
      1760       1770       1780       1790       1800
LADKHFNYTS LIAFHCKRGV SMGTPKLLRT SECDFVFEWE TPVVCPDEVR   ← Glycosite 2126 (7) [@1757]
      1810       1820       1830       1840       1850
MDGCTLTDEQ LLYSFNLSSL STSTFKVTRD SRTYSVGVCT FAVGPEQGGC   ← Glycosite 3743 (9) [@N1826]
      1860       1870       1880       1890       1900
KDGGVCLLSG TKGASFGRLQ SMKLDYRHQD EAVVLSYVNG DRCPPETDDG
      1910       1920       1930       1940       1950
VPCVFPFIFN GKSYEECIIE SRAKLWCSTT ADYDRDHEWG FCRHSNSYRT
      1960       1970       1980       1990       2000
SSIIFKCDED EDIGRPQVFS EVRGCDVTFE WKTKVVCPPK KLECKFVQKH
      2010       2020       2030       2040       2050
KTYDLRLLSS LTGSWSLVHN GVSYYINLCQ KIYKGPLGCS ERASICRRTT
      2060       2070       2080       2090       2100
TGDVQVLGLV HTQKLGVIGD KVVVTYSKGY PCGGNKTASS VIELTCTKTV
      2110       2120       2130       2140       2150
GRPAFKRFDI DSCTYYFSWD SRAACAVKPQ EVQMVNGTIT NPINGKSFSL
      2160       2170       2180       2190       2200
GDIYFKLFRA SGDMRTNGDN YLYEIQLSSI TSSRNPACSG ANICQVKPND
      2210       2220       2230       2240       2250
QHFSRKVGTS DKTKYYLQDG DLDVVFASSS KCGKDKTKSV SSTIFFHCDP
      2260       2270       2280       2290       2300
LVEDGIPEFS HETADCQYLF SWYTSAVCPL GVGFDSENPG DDGQMHKGLS
      2310       2320       2330       2340       2350
ERSQAVGAVL SLLLVALTCC LLALLLYKKE RRETVISKLT TCCRRSSNVS
      2360       2370       2380       2390       2400
YKYSKVNKEE ETDENETEWL MEEIQLPPPR QGKEGQENGH ITTKSVKALS
      2410       2420       2430       2440       2450
SLHGDDQDSE DEVLTIPEVK VHSGRGAGAE SSHPVRNAQS NALQEREDDR
      2460       2470       2480       2490
VGLVRGEKAR KGKSSSAQQK TVSSTKLVSF HDDSDEDLLH
```

LRP1 (Prolow-density lipoprotein receptor-related protein 1)

Data ID[a] (Glycosite #)[b]	Peptide sequence	Ratio of sialoglycosite abundance in 1,3,4-O-Bu_3ManNAc-treated to control cells: MCF10A	T-47D	MDA-MB-231
**3357[a] (1)[b]**	LNGTDPIVAADSK	0.10	32.79	22.55
**3721 (2)**	LYWISSGNHTINR	2.20	0.32	2.42
**6124 (3)**	WTGHNVTVVQR	2.20	0.32	2.42
**3766 (4)**	MHLNGSNVQVLHR	0.73	16.15	2.08
**826 (5)**	DNATDSVPLR	0.69	1.42	1.25
**841 (6)**	DNTTCYEFK	0.53	1.19	1.20
**563 (7)**	CNASSQFLCSSGR	0.73	1.58	1.19
**2323 (8)**	IETILLNGTDR	0.88	1.49	1.17
**1569 (9)**	FNSTEYQVVTR	0.92	1.80	1.12
**5073 (10)**	TCVSNCTASQFVCK	3.20	1.69	0.98
**3588 (11)**	LTSCATNASICGDEAR	0.07	2.06	0.92
**5530 (12)**	VDIPQQPMGIIAVANDTNSCELSPCR	0.45	4.35	0.37

[a] The data ID number is provided in **Column A** of **Sheet 1** of **Supplemental File #3**

[b] The glycosite # represents the labeling scheme used in **Figure 4** of the main text

Amino acid sequence (*from* Uniprot; https://www.uniprot.org/uniprot/Q07954)

N-glycan consensus sequons in **red** are predicted by NetNGly to be occupied

N-glycan consensus sequons in **blue** are predicted by NetNGly to be NOT occupied

Sequences underlined using a dotted line represent peptide sequences identified as "glycosites"

```
        10         20         30         40         50
MLTPPLLLLL PLLSALVAAA IDAPKTCSPK QFACRDQITC ISKGWRCDGE
        60         70         80         90        100
RDCPDGSDEA PEICPQSKAQ RCQPNEHNCL GTELCVPMSR LCNGVQDCMD
       110        120        130        140        150
GSDEGPHCRE LQGNCSRLGC QHHCVPTLDG PTCYCNSSFQ LQADGKTCKD
       160        170        180        190        200
FDECSVYGTC SQLCTNTDGS FICGCVEGYL LQPDNRSCKA KNEPVDRPPV
       210        220        230        240        250
LLIANSQNIL ATYLSGAQVS TITPTSTRQT TAMDFSYANE TVCWVHVGDS
       260        270        280        290        300
AAQTQLKCAR MPGLKGFVDE HTINISLSLH HVEQMAIDWL TGNFYFVDDI
       310        320        330        340        350
DDRIFVCNRN GDTCVTLLDL ELYNPKGIAL DPAMGKVFFT DYGQIPKVER
       360        370        380        390        400
CDMDGQNRTK LVDSKIVFPH GITLDLVSRL VYWADAYLDY IEVVDYEGKG
       410        420        430        440        450
RQTIIQGILI EHLYGLTVFE NYLYATNSDN ANAQQKTSVI RVNRFNSTEY   ← Glycosite 1569 (9) [@N446]
       460        470        480        490        500
QVVTRVDKGG ALHIYHQRRQ PRVRSHACEN DQYGKPGGCS DICLLANSHK
       510        520        530        540        550
ARTCRCRSGF SLGSDGKSCK KPEHELFLVY GKGRPGIIRG MDMGAKVPDE
       560        570        580        590        600
HMIPIENLMN PRALDFHAET GFIYFADTTS YLIGRQKIDG TERETILKDG
       610        620        630        640        650
IHNVEGVAVD WMGDNLYWTD DGPKKTISVA RLEKAAQTRK TLIEGKMTHP
       660        670        680        690        700
RAIVVDPLNG WMYWTDWEED PKDSRRGRLE RAWMDGSHRD IFVTSKTVLW
       710        720        730        740        750
PNGLSLDIPA GRLYWVDAFY DRIETILLNG TDRKIVYEGP ELNHAFGLCH   ← Glycosite 2523 (8) [@N729]
       760        770       780         790        800
HGNYLFWTEY RSGSVYRLER GVGGAPPTVT LLRSERPPIF EIRMYDAQQQ
       810        820        830        840        850
QVGTNKCRVN NGGCSSLCLA TPGSRQCACA EDQVLDADGV TCLANPSYVP
       860        870        880        890        900
PPQCQPGEFA CANSRCIQER WKCDGDNDCL DNSDEAPALC HQHTCPSDRF
       910        920        930        940        950
KCENNRCIPN RWLCDGDNDC GNSEDESNAT CSARTCPPNQ FSCASGRCIP
       960        970        980        990       1000
ISWTCDLDDD CGDRSDESAS CAYPTCFPLT QFTCNNGRCI NINWRCDNDN
      1010       1020       1030       1040       1050
DCGDNSDEAG CSHSCSSTQF KCNSGRCIPE HWTCDGDNDC GDYSDETHAN
      1060       1070       1080       1090       1100
CTNQATRPPG GCHTDEFQCR LDGLCIPLRW RCDGDTDCMD SSDEKSCEGV
      1110       1120       1130       1140       1150
THVCDPSVKF GCKDSARCIS KAWVCDGDND CEDNSDEENC ESLACRPPSH
      1160       1170       1180       1190       1200
PCANNTSVCL PPDKLCDGND DCGDGSDEGE LCDQCSLNNG GCSHNCSVAP
      1210       1220       1230       1240       1250
GEGIVCSCPL GMELGPDNHT CQIQSYCAKH LKCSQKCDQN KFSVKCSCYE
      1260       1270       1280       1290       1300
GWVLEPDGES CRSLDPFKPF IIFSNRHEIR RIDLHKGDYS VLVPGLRNTI
      1310       1320       1330       1340       1350
ALDFHLSQSA LYWTDVVEDK IYRGKLLDNG ALTSFEVVIQ YGLATPEGLA
      1360       1370       1380       1390       1400
VDWIAGNIYW VESNLDQIEV AKLDGTLRTT LLAGDIEHPR AIALDPRDGI
      1410       1420       1430       1440       1450
LFWTDWDASL PRIEAASMSG AGRRTVHRET GSGGWPNGLT VDYLEKRILW
      1460       1470       1480       1490       1500
IDARSDAIYS ARYDGSGHME VLRGHEFLSH PFAVTLYGGE VYWTDWRTNT
      1510       1520       1530       1540       1550
LAKANKWTGH NVTVVQRTNT QPFDLQVYHP SRQPMAPNPC EANGGQGPCS   ← Glycosite 6124 (3) [@N1511]
     1560        1570       1580       1590       1600
HLCLINYNRT VSCACPHLMK LHKDNTTCYE FKKFLLYARQ MEIRGVDLDA   ← Glycosite 841 (6) [@N1575]
      1610       1620       1630       1640       1650
PYYNYIISFT VPDIDNVTVL DYDAREQRVY WSDVRTQAIK RAFINGTGVE
      1660       1670       1680       1690       1700
TVVSADLPNA HGLAVDWVSR NLFWTSYDTN KKQINVARLD GSFKNAVVQG
      1710       1720       1730       1740       1750
LEQPHGLVVH PLRGKLYWTD GDNISMANMD GSNRTLLFSG QKGPVGLAID
      1760       1770       1780      1790        1800
FPESKLYWIS SGNHTINRCN LDGSGLEVID AMRSQLGKAT ALAIMGDKLW   ← Glycosite 3721 (2) [@N1763]
      1810       1820       1830       1840       1850
WADQVSEKMG TCSKADGSGS VVLRNSTTLV MHMKVYDESI QLDHKGTNPC
      1860       1870       1880       1890       1900
SVNNGDCSQL CLPTSETTRS CMCTAGYSLR SGQQACEGVG SFLLYSVHEG
      1910       1920       1930       1940       1950
IRGIPLDPND KSDALVPVSG TSLAVGIDFH AENDTIYWVD MGLSTISRAK
      1960       1970       1980        1990       2000
RDQTWREDVV TNGIGRVEGI AVDWIAGNIY WTDQGFDVIE VARLNGSFRY
      2010       2020       2030       2040       2050
VVISQGLDKP RAITVHPEKG YLFWTEWGQY PRIERSRLDG TERVVLVNVS
      2060       2070       2080       2090       2100
ISWPNGISVD YQDGKLYWCD ARTDKIERID LETGENREVV LSSNNMDMFS
      2110       2120       2130       2140       2150
VSVFEDFIYW SDRTHANGSI KRGSKDNATD SVPLRTGIGV QLKDIKVFNR   ← Glycosite 826 (5) [@N2127]
      2160       2170       2180       2190       2200
DRQKGTNVCA VANGGCQQLC LYRGRGQRAC ACAHGMLAED GASCREYAGY
      2210       2220       2230       2240       2250
LLYSERTILK SIHLSDERNL NAPVQPFEDP EHMKNVIALA FDYRAGTSPG
      2260       2270       2280       2290       2300
TPNRIFFSDI HFGNIQQIND DGSRRITIVE NVGSVEGLAY HRGWDTLYWT
      2310       2320       2330       2340       2350
SYTTSTITRH TVDQTRPGAF ERETVITMSG DDHPRAFVLD ECQNLMFWTN
      2360       2370       2380       2390       2400
WNEQHPSIMR AALSGANVLT LIEKDIRTPN GLAIDHRAEK LYFSDATLDK
      2410       2420       2430       2440       2450
IERCEYDGSH RYVILKSEPV HPFGLAVYGE HIFWTDWVRR AVQRANKHVG
      2460       2470       2480       2490       2500
SNMKLLRVDI PQQPMGIIAV ANDTNSCELS PCRINNGGCQ DLCLLTHQGH   ← Glycosite 5530 (12) [@N2472]
      2510       2520       2530       2540       2550
VNCSCRGGRI LQDDLTCRAV NSSCRAQDEF ECANGECINF SLTCDGVPHC
      2560       2570       2580       2590       2600
KDKSDEKPSY CNSRRCKKTF RQCSNGRCVS NMLWCNGADD CGDGSDEIPC
      2610       2620       2630       2640       2650
NKTACGVGEF RCRDGTCIGN SSRCNQFVDC EDASDEMNCS ATDCSSYFRL
      2660       2670       2680       2690       2700
GVKGVLFQPC ERTSLCYAPS WVCDGANDCG DYSDERDCPG VKRPRCPLNY
      2710       2720       2730       2740       2750
FACPSGRCIP MSWTCDKEDD CEHGEDETHC NKFCSEAQFE CQNHRCISKQ
      2760       2770       2780       2790       2800
WLCDGSDDCG DGSDEAAHCE GKTCGPSSFS CPGTHVCVPE RWLCDGDKDC
      2810       2820       2830       2840       2850
ADGADESIAA GCLYNSTCDD REFMCQNRQC IPKHFVCDHD RDCADGSDES
      2860       2870       2880       2890       2900
PECEYPTCGP SEFRCANGRC LSSRQWECDG ENDCHDQSDE APKNPHCTSQ
      2910       2920       2930       2940       2950
EHKCNASSQF LCSSGRCVAE ALLCNGQDDC GDSSDERGCH INECLSRKLS   ← Glycosite 563 (7) [@N2905]
      2960       2970       2980       2990       3000
GCSQDCEDLK IGFKCRCRPG FRLKDDGRTC ADVDECSTTF PCSQRCINTH
      3010       3020       3030       3040       3050
GSYKCLCVEG YAPRGGDPHS CKAVTDEEPF LIFANRYYLR KLNLDGSNYT
      3060       3070       3080       3090       3100
LLKQGLNNAV ALDFDYREQM IYWTDVTTQG SMIRRMHLNG SNVQVLHRTG   ← Glycosite 3766 (4) [@N3089]
      3110       3120       3130       3140       3150
LSNPDGLAVD WVGGNLYWCD KGRDTIEVSK LNGAYRTVLV SSGLREPRAL
      3160       3170       3180       3190       3200
VVDVQNGYLY WTDWGDHSLI GRIGMDGSSR SVIVDTKITW PNGLTLDYVT
      3210       3220       3230       3240       3250
ERIYWADARE DYIEFASLDG SNRHVVLSQD IPHIFALTLF EDYVYWTDWE
      3260       3270       3280       3290       3300
TKSINRAHKT TGTNKTLLIS TLHRPMDLHV FHALRQPDVP NHPCKVNNGG
      3310       3320       3330       3340       3350
CSNLCLLSPG GGHKCACPTN FYLGSDGRTC VSNCTASQFV CKNDKCIPFW   ← Glycosite 5073 (10) [@N3333]
      3360       3370       3380       3390       3400
WKCDTEDDCG DHSDEPPDCP EFKCRPGQFQ CSTGICTNPA FICDGDNDCQ
      3410       3420       3430       3440       3450
DNSDEANCDI HVCLPSQFKC TNTNRCIPGI FRCNGQDNCG DGEDERDCPE
      3460       3470       3480       3490       3500
VTCAPNQFQC SITKRCIPRV WVCDRDNDCV DGSDEPANCT QMTCGVDEFR
      3510       3520       3530       3540       3550
CKDSGRCIPA RWKCDGEDDC GDGSDEPKEE CDERTCEPYQ FRCKNNRCVP
      3560       3570       3580       3590       3600
GRWQCDYDND CGDNSDEESC TPRPCSESEF SCANGRCIAG RWKCDGDHDC
      3610       3620       3630       3640       3650
ADGSDEKDCT PRCDMDQFQC KSGHCIPLRW RCDADADCMD GSDEEACGTG
      3660       3670       3680       3690       3700
VRTCPLDEFQ CNNTLCKPLA WKCDGEDDCG DNSDENPEEC ARFVCPPNRP
      3710       3720       3730       3740       3750
FRCKNDRVCL WIGRQCDGTD NCGDGTDEED CEPPTAHTTH CKDKKEFLCR
      3760       3770       3780       3790       3800
NQRCLSSSLR CNMFDDCGDG SDEEDCSIDP KLTSCATNAS ICGDEARCVR   ← Glycosite 3588 (11) [@N3788]
      3810       3820       3830       3840       3850
TEKAAYCACR SGFHTVPGQP GCQDINECLR FGTCSQLCNN TKGGHLCSCA
      3860       3870       3880       3890       3900
RNFMKTHNTC KAEGSEYQVL YIADDNEIRS LFPGHPHSAY EQAFQGDESV
      3910       3920       3930       3940       3950
RIDAMDVHVK AGRVYWTNWH TGTISYRSLP PAAPPTTSNR HRRQIDRGVT
      3960       3970       3980       3990       4000
HLNISGLKMP RGIAIDWVAG NVYWTDSGRD VIEVAQMKGE NRKTLISGMI
      4010       4020       4030       4040       4050
DEPHAIVVDP LRGTMYWSDW GNHPKIETAA MDGTLRETLV QDNIQWPTGL
      4060       4070       4080       4090       4100
AVDYHNERLY WADAKLSVIG SIRLNGTDPI VAADSKRGLS HPFSIDVFED
      4110       4120       4130       4140       4150
YIYGVTYINN RVFKIHKFGH SPLVNLTGGL SHASDVVLYH QHKQPEVTNP
      4160       4170       4180       4190       4200
CDRKKCEWLC LLSPSGPVCT CPNGKRLDNG TCVPVPSPTP PPDAPRPGTC
      4210       4220       4230       4240       4250
NLQCFNGGSC FLNARRQPKC RCQPRYTGDK CELDQCWEHC RNGGTCAASP
      4260         4270       4280       4290       4300
SGMPTCRCPT GFTGPKCTQQ VCAGYCANNS TCTVNQGNQP QCRCLPGFLG
      4310       4320       4330       4340       4350
DRCQYRQCSG YCENFGTCQM AADGSRQCRC TAYFEGSRCE VNKCSRCLEG
      4360       4370       4380       4390      4400
ACVVNKQSGD VTCNCTDGRV APSCLTCVGH CSNGGSCTMN SKMMPECQCP
      4410       4420       4430       4440       4450
PHMTGPRCEE HVFSQQQPGH IASILIPLLL LLLLVLVAGV VFWYKRRVQG
      4460       4470       4480       4490       4500
AKGFQHQRMT NGAMNVEIGN PTYKMYEGGE PDDVGGLLDA DFALDPDKPT
      4510       4520       4530       4540
NFTNPVYATL YMGGHGSRHS LASTDEKREL LGRGPEDEIG DPLA
```

NCSTN (Nicastrin)

Data ID[a] (Glycosite #)[b]	Peptide sequence	Ratio of sialoglycosite abundance in 1,3,4-O-Bu_3ManNAc-treated to control cells: MCF10A	T-47D	MDA-MB-231
820[a] (1)[b]	DLYEYSWVQGPLHS**NET**DRLPR	0.61	11.50	1.29
345 (2)	A**NNS**WFQSILR	0.69	34.8	1.25
3283 (3)	LL**NAT**HQIGCQSSISGDTGVIHVVEK	260.17	0.95	1.13
3999 (4)	**NIS**GVVLADHSGAFHNK	1.37	2.24	1.12
4575 (5)	RP**NQS**QPLPPSSLQR	4.83	1.73	1.02
314 (6)	ALYELAGGT**NFS**DTVQADPQTVTR	36.57	2.03	0.37

[a] The data ID number is provided in **Column A** of **Sheet 1** of **Supplemental File #3**

[b] The glycosite # represents the labeling scheme used in **Figure 4** of the main text

Amino acid sequence (*from* Uniprot; https://www.uniprot.org/uniprot/Q92542)

N-glycan consensus sequons in **red** are predicted by NetNGly to be occupied

N-glycan consensus sequons in **blue** are predicted by NetNGly to be NOT occupied

Sequences underlined using a dotted line represent peptide sequences identified as "glycosites"

```
        10         20         30         40         50
MATAGGGSGA DPGSRGLLRL LSFCVLLAGL CRGNSVERKI YIPLNKTAPC
        60         70         80         90        100
VRLLNATHQI GCQSSISGDT GVIHVVEKEE DLQWVLTDGP NPPYMVLLES
       110        120        130        140        150
KHFTRDLMEK LKGRTSRIAG LAVSLTKPSP ASGFSPSVQC PNDGFGVYSN
       160        170        180        190        200
SYGPEFAHCR EIQWNSLGNG LAYEDFSFPI FLLEDENETK VIKQCYQDHN
       210        220        230        240        250
LSQNGSAPTF PLCAMQLFSH MHAVISTATC MRRSSIQSTF SINPEIVCDP
       260        270        280        290        300
LSDYNVWSML KPINTTGTLK PDDRVVVAAT RLDSRSFFWN VAPGAESAVA
       310        320        330        340        350
SFVTQLAAAE ALQKAPDVTT LPRNVMFVFF QGETFDYIGS SRMVYDMEKG
       360        370        380        390        400
KFPVQLENVD SFVELGQVAL RTSLELWMHT DPVSQKNESV RNQVEDLLAT
       410        420        430        440        450
LEKSGAGVPA VILRRPNQSQ PLPPSSLQRF LRARNISGVV LADHSGAFHN
       460        470        480        490        500
KYYQSIYDTA ENINVSYPEW LSPEEDLNFV TDTAKALADV ATVLGRALYE
       510        520        530        540        550
LAGGTNFSDT VQADPQTVTR LLYGFLIKAN NSWFQSILRQ DLRSYLGDGP
       560        570        580        590        600
LQHYIAVSSP TNTTYVVQYA LANLTGTVVN LTREQCQDPS KVPSENKDLY
       610        620        630        640        650
EYSWVQGPLH SNETDRLPRC VRSTARLARA LSPAFELSQW SSTEYSTWTE
       660        670        680        690        700
SRWKDIRARI FLIASKELEL ITLTVGFGIL IFSLIVTYCI NAKADVLFIA

PREPGAVSY
```

← **Glycosite 3283 (3)** [@N55]

Glycosite 4575 (5) [@N417]

Glycosite 3999 (4) [@N435]

Glycosite 314 (6) [@N516]

Glycosite 345 (2) [@N530]

Glycosite 820 (1) [@N612]

PLXNB2 (Plexin-B2)

Data ID[a] (Glycosite #)[b]	Peptide sequence	Ratio of sialoglycosite abundance in 1,3,4-O-Bu_3ManNAc-treated to control cells: MCF10A	T-47D	MDA-MB-231
**299[a] (1)[b]**	ALS**NIS**LR	1.43	2.16	66.84
**3509 (2)**	LSHDA**NET**LPLHLYVK	176.44	2.16	4.66
**579 (3)**	CVYEALC**NTT**SECPPPVITR	3.85	2.20	2.70
**4767 (4)**	SI**NVT**GQGFSLIQR	1.51	2.23	1.34
**988 (5)**	EAESLQPMTVVGTDYVFH**NDT**K	3.52	3.38	1.21
**5091 (6)**	TEAGAFEYVPDPTFE**NFT**GGVK	151.77	2.10	1.09
**1799 (7)**	GGL**NLT**AVTVAAEN**NHT**VAFLGTSDGR	1.41	2.24	1.06
**4635 (8)**	SCVAVTSAQPQ**NMS**R	1.21	0.32	0.89
**3133 (9)**	LHVTLY**NCS**FGR	1.51	2.61	0.65
**5995 (10)**	VVFLSPAVPEEPEAY**NLT**VLIEMDGHR	2.53	1.54	0.53

[a] The data ID number is provided in **Column A** of **Sheet 1** of **Supplemental File #3**

[b] The glycosite # represents the labeling scheme used in **Figure 4** of the main text

Amino acid sequence (*from* Uniprot; https://www.uniprot.org/uniprot/O15031)

N-glycan consensus sequons in **red** are predicted by NetNGly to be occupied

N-glycan consensus sequons in **blue** are predicted by NetNGly to be NOT occupied

Sequences underlined using a dotted line represent peptide sequences identified as "glycosites"

```
        10         20         30         40         50
MALQLWALTL LGLLGAGASL RPRKLDFFRS EKELNHLAVD EASGVVYLGA
        60         70         80         90        100
VNALYQLDAK LQLEQQVATG PALDNKKCTP PIEASQCHEA EMTDNVNQLL
       110        120        130        140        150
LLDPPRKRLV ECGSLFKGIC ALRALSNISL RLFYEDGSGE KSFVASNDEG  ← Glycosite 299 (1) [@N127]
       160        170        180        190        200
VATVGLVSST GPGGDRVLFV GKGNGPHDNG IIVSTRLLDR TDSREAFEAY
       210        220        230        240        250
TDHATYKAGY LSTNTQQFVA AFEDGPYVFF VFNQQDKHPA RNRTLLARMC
       260        270        280        290        300
REDPNYYSYL EMDLQCRDPD IHAAAFGTCL AASVAAPGSG RVLYAVFSRD
       310        320        330        340        350
SRSSGGPGAG LCLFPLDKVH AKMEANRNAC YTGTREARDI FYKPFHGDIQ
       360        370        380        390        400
CGGHAPGSSK SFPCGSEHLP YPLGSRDGLR GTAVLQRGGL NLTAVTVAAE  ← Glycosite 1799 (7) [@N390 or N402]
       410        420        430        440        450      (has 2 possible glycan sites)
NNHTVAFLGT SDGRILKVYL TPDGTSSEYD SILVEINKRV KRDLVLSGDL
       460        470        480        490        500
GSLYAMTQDK VFRLPVQECL SYPTCTQCRD SQDPYCGWCV VEGRCTRKAE
       510        520        530        540        550
CPRAEEASHW LWSRSKSCVA VTSAQPQNMS RRAQGEVQLT VSPLPALSEE  ← Glycosite 4635 (8) [@N528]
       560        570        580        590        600
DELLCLFGES PPHPARVEGE AVICNSPSSI PVTPPGQDHV AVTIQLLLRR
       610        620        630        640        650
GNIFLTSYQY PFYDCRQAMS LEENLPCISC VSNRWTCQWD LRYHECREAS
       660        670        680        690        700
PNPEDGIVRA HMEDSCPQFL GPSPLVIPMN HETDVNFQGK NLDTVKGSSL
       710        720        730        740        750
HVGSDLLKFM EPVTMQESGT FAFRTPKLSH DANETLPLHL YVKSYGKNID  ← Glycosite 3509 (2) [@N733]
       760        770        780        790        800
SKLHVTLYNC SFGRSDCSLC RAANPDYRCA WCGGQSRCVY EALCNTTSEC  ← Glycosite 3133 (9) [@N759]
       810        820        830        840        850
PPPVITRIQP ETGPLGGGIR ITILGSNLGV QAGDIQRISV AGRNCSFQPE  ↖ Glycosite 579 (3) [@N795]
       860        870        880        890        900
RYSVSTRIVC VIEAAETPFT GGVEVDVFGK LGRSPPNVQF TFQQPKPLSV
       910        920        930        940        950
EPQQGPQAGG TTLTIHGTHL DTGSQEDVRV TLNGVPCKVT KFGAQLQCVT
       960        970        980        990       1000
GPQATRGQML LEVSYGGSPV PNPGIFFTYR ENPVLRAFEP LRSFASGGRS  ← Glycosite 4767 (4) [@ N1002]
      1010       1020       1030       1040       1050
INVTGQGFSL IQRFAMVVIA EPLQSWQPPR EAESLQPMTV VGTDYVFHND  ← Glycosite 988 (5) [@ N1049]
      1060       1070       1080       1090       1100
TKVVFLSPAV PEEPEAYNLT VLIEMDGHRA LLRTEAGAFE YVPDPTFENF  ← Glycosite 5995 (10) [@ N1068]
      1110       1120       1130       1140       1150
TGGVKKQVNK LIHARGTNLN KAMTLQEAEA FVGAERCTMK TLTETDLYCE  ↖ Glycosite 5091 (6) [@ N1099]
      1160       1170       1180       1190       1200
PPEVQPPPKR RQKRDTTHNL PEFIVKFGSR EWVLGRVEYD TRVSDVPLSL
      1210       1220       1230       1240       1250
ILPLVIVPMV VVIAVSVYCY WRKSQQAERE YEKIKSQLEG LEESVRDRCK
      1260       1270       1280       1290       1300
KEFTDLMIEM EDQTNDVHEA GIPVLDYKTY TDRVFFLPSK DGDKDVMITG
      1310       1320       1330       1340       1350
KLDIPEPRRP VVEQALYQFS NLLNSKSFLI NFIHTLENQR EFSARAKVYF
      1360       1370       1380       1390       1400
ASLLTVALHG KLEYYTDIMH TLFLELLEQY VVAKNPKLML RRSETVVERM
      1410       1420       1430       1440       1450
LSNWMSICLY QYLKDSAGEP LYKLFKAIKH QVEKGPVDAV QKKAKYTLND
      1460       1470       1480       1490       1500
TGLLGDDVEY APLTVSVIVQ DEGVDAIPVK VLNCDTISQV KEKIIDQVYR
      1510       1520       1530       1540       1550
GQPCSCWPRP DSVVLEWRPG STAQILSDLD LTSQREGRWK RVNTLMHYNV
      1560       1570       1580       1590       1600
RDGATLILSK VGVSQQPEDS QQDLPGERHA LLEEENRVWH LVRPTDEVDE
      1610       1620       1630       1640       1650
GKSKRGSVKE KERTKAITEI YLTRLLSVKG TLQQFVDNFF QSVLAPGHAV
      1660       1670       1680       1690       1700
PPAVKYFFDF LDEQAEKHNI QDEDTIHIWK TNSLPLRFWV NILKNPHFIF
      1710       1720       1730       1740       1750
DVHVHEVVDA SLSVIAQTFM DACTRTEHKL SRDSPSNKLL YAKEISTYKK
      1760       1770       1780       1790       1800
MVEDYYKGIR QMVQVSDQDM NTHLAEISRA HTDSLNTLVA LHQLYQYTQK
      1810       1820       1830
YYDEIINALE EDPAAQKMQL AFRLQQIAAA LENKVTDL
```

PLXND1 (Plexin-D1)

Data ID[a] (Glycosite #)[b]	Peptide sequence	Ratio of sialoglycosite abundance in 1,3,4-O-Bu_3ManNAc-treated to control cells: MCF10A	T-47D	MDA-MB-231
**5774 (1)**	VL**NST**LITCPSPGALS**NAS**APVDFFINGR	1.92	7.18	1.44
**3716 (2)**	LYQLSGA**NLS**LEAEAAVGPVPDSPLCHAPQLPQASCEHPR	89.12	1.60	1.41
**333 (3)**	A**NFT**IYDCSR	1.32	3.48	0.65
**6383 (4)**	YTVSEEIVCVTGPAPGPLSGVVTV**NAS**K	160.64	27.62	0.65
**1466 (5)**	FHMVQ**NVS**MAVHHIGR	12.97	1.79	0.64

[a] The data ID number is provided in **Column A** of **Sheet 1** of **Supplemental File #3**

[b] The glycosite # represents the labeling scheme used in **Figure 4** of the main text

Amino acid sequence (*from* Uniprot; https://www.uniprot.org/uniprot/Q9Y4D7)

N-glycan consensus sequons in **red** are predicted by NetNGly to be occupied

N-glycan consensus sequons in **blue** are predicted by NetNGly to be NOT occupied

Sequences underlined using a dotted line represent peptide sequences identified as "glycosites"

```
        10         20         30         40         50
MAPRAAGGAP LSARAAAASP PPFQTPPRCP VPLLLLLLLG AARAGALEIQ
        60         70         80         90        100
RRFPSPTPTN NFALDGAAGT VYLAAVNRLY QLSGANLSLE AEAAVGPVPD   <- Glycosite 3716 (2) [@N86]
       110        120        130        140        150
SPLCHAPQLP QASCEHPRRL TDNYNKILQL DPGQGLVVVC GSIYQGFCQL
       160        170        180        190        200
RRRGNISAVA VRFPPAAPPA EPVTVFPSML NVAANHPNAS TVGLVLPPAA
       210        220        230        240        250
GAGGSRLLVG ATYTGYGSSF FPRNRSLEDH RFENTPEIAI RSLDTRGDLA
       260        270        280        290        300
KLFTFDLNPS DDNILKIKQG AKEQHKLGFV SAFLHPSDPP PGAQSYAYLA
       310        320        330        340        350
LNSEARAGDK ESQARSLLAR ICLPHGAGGD AKKLTESYIQ LGLQCAGGAG
       360        370        380        390        400
RGDLYSRLVS VFPARERLFA VFERPQGSPA ARAAPAALCA FRFADVRAAI
       410        420        430        440        450
RAARTACFVE PAPDVVAVLD SVVQGTGPAC ERKLNIQLQP EQLDCGAAHL
       460        470        480        490        500
QHPLSILQPL KATPVFRAPG LTSVAVASVN NYTAVFLGTV NGRLLKINLN
       510        520        530        540        550
ESMQVVSRRV VTVAYGEPVH HVMQFDPADS GYLYLMTSHQ MARVKVAACN
       560        570        580        590        600
VHSTCGDCVG AADAYCGWCA LETRCTLQQD CTNSSQHFW TSASEGPSRC
       610        620        630        640        650
PAMTVLPSEI DVRQEYPGMI LQISGSLPSL SGMEMACDYG NNIRTVARVP
       660        670        680        690        700
GPAFGHQIAY CNLLPRDQFP PFPPNQDHVT VEMSVRVNGR NIVKANFTIY   <- Glycosite 333 (3) [@N696]
       710        720        730        740        750
DCSRTAQVYP HTACTSCLSA QWPCFWCSQQ HSCVSNQSRC EASPNPTSPQ
       760        770        780        790        800
DCPRTLLSPL APVPTGGSQN ILVPLANTAF FQGAALECSF GLEEIFEAVW
       810        820        830        840        850
VNESVVRCDQ VVLHTTRKSQ VFPLSLQLKG RPARFLDSPE PMTVMVYNCA
       860        870        880        890        900
MGSPDCSQCL GREDLGHLCM WSDGCRLRGP LQPMAGTCPA PEIHAIEPLS
       910        920        930        940        950
GPLDGGTLLT IRGRNLGRRL SDVAHGVWIG GVACEPLPDR YTVSEEIVCV
       960        970        980        990       1000
TGPAPGPLSG VVTVNASKEG KSRDRFSYVL PLVHSLEPTM GPKAGGTRIT   <- Glycosite 6383 (4) [@N965]
      1010       1020       1030       1040       1050
IHGNDLHVGS ELQVLVNDTD PCTELMRTDT SIACTMPEGA LPAPVPVCVR
      1060       1070       1080       1090       1100
FERRGCVHGN LTFWYMQNPV ITAISPRRSP VSGGRTITVA GERFHMVQNV   <- Glycosite 1466 (5) [@N1099]
      1110       1120       1130       1140       1150
SMAVHHIGRE PTLCKVLNST LITCPSPGAL SNASAPVDFF INGRAYADEV   <- Glycosite 5774 (1)
                                                            [two possible sites @ N1118 and N113
      1160       1170       1180       1190       1200
AVAEELLDPE EAQRGSRFRL DYLPNPQFST AKREKWIKHH PGEPLTLVIH
      1210       1220       1230       1240       1250
KEQDSLGLQS HEYRVKIGQV SCDIQIVSDR IIHCSVNESL GAAVGQLPIT
      1260       1270       1280       1290       1300
IQVGNFNQTI ATLQLGGSET AIIVSIVICS VLLLLSVVAL FVFCTKSRRA
      1310       1320       1330       1340       1350
ERYWQKTLLQ MEEMESQIRE EIRKGFAELQ TDMTDLTKEL NRSQGIPFLE
      1360       1370       1380       1390       1400
YKHFVTRTFF PKCSSLYEER YVLPSQTLNS QGSSQAQETH PLLGEWKIPE
      1410       1420       1430       1440       1450
SCRPNMEEGI SLFSSLLNNK HFLIVFVHAL EQQKDFAVRD RCSLASLLTI
      1460       1470       1480       1490       1500
ALHGKLEYYT SIMKELLVDL IDASAAKNPK LMLRRTESVV EKMLTNWMSI
      1510       1520       1530       1540       1550
CMYSCLRETV GEPFFLLLCA IKQQINKGSI DAITGKARYT LSEEWLLREN
      1560       1570       1580       1590       1600
IEAKPRNLNV SFQGCGMDSL SVRAMDTDTL TQVKEKILEA FCKNVPYSQW
      1610       1620       1630       1640       1650
PRAEDVDLEW FASSTQSYIL RDLDDTSVVE DGRKKLNTLA HYKIPEGASL
      1660       1670       1680       1690       1700
AMSLIDKKDN TLGRVKDLDT EKYFHLVLPT DELAEPKKSH RQSHRKKVLP
      1710       1720       1730       1740       1750
EIYLTRLLST KGTLQKFLDD LFKAILSIRE DKPPLAVKYF FDFLEEQAEK
      1760       1770       1780       1790       1800
RGISDPDTLH IWKTNSLPLR FWVNILKNPQ FVFDIDKTDH IDACLSVIAQ
      1810       1820       1830       1840       1850
AFIDACSISD LQLGKDSPTN KLLYAKEIPE YRKIVQRYYK QIQDMTPLSE
      1860       1870       1880       1890       1900
QEMNAHLAEE SRKYQNEFNT NVAMAEIYKY AKRYRPQIMA ALEANPTARR
      1910       1920
TQLQHKFEQV VALMEDNIYE CYSEA
```

FAT1 (Protocadherin Fat 1;Protocadherin Fat 1, nuclear form)

Data ID[a] (Glycosite #)[b]	Peptide sequence	Ratio of sialoglycosite abundance in 1,3,4-O-Bu_3ManNAc-treated to control cells: MCF10A	T-47D	MDA-MB-231
4485[a] (1)[b]	QVTQEML**NHT**IAIR	1.32	5.94	19.30
6379 (2)	YTL**NIT**VYDLGIPQK	2.14	10.28	5.38
2933 (3)	LDIGQYLL**NVS**VTDGK	0.29	1.14	1.72
5142 (4)	TGALTVQ**NTT**QLR	3.00	0.51	1.64
4491 (5)	QVY**NLT**VR	0.68	0.50	1.26
2557 (6)	I**NSS**VTDIEEIIGVR	2.52	2.63	0.75
5857 (7)	V**NTT**TVNIDVSDVNDNAPVFSR	2.63	0.83	0.06

[a] The data ID number is provided in **Column A** of **Sheet 1** of **Supplemental File #3**

[b] The glycosite # represents the labeling scheme used in **Figure 4** of the main text

Amino acid sequence (*from* Uniprot; https://www.uniprot.org/uniprot/Q14517)

N-glycan consensus sequons in **red** are predicted by NetNGly to be occupied

N-glycan consensus sequons in **blue** are predicted by NetNGly to be NOT occupied

Sequences underlined using a dotted line represent peptide sequences identified as "glycosites"

```
        10         20         30         40         50
MGRHLALLLL LLLLFQHFGD SDGSQRLEQT PLQFTHLEYN VTVQENSAAK
        60          70         80         90        100
TYVGHPVKMG VYITHPAWEV RYKIVSGDSE NLFKAEEYIL GDFCFLRIRT
       110        120        130        140        150
KGGNTAILNR EVKDHYTLIV KALEKNTNVE ARTKVRVQVL DTNDLRPLFS
       160        170        180        190        200
PTSYSVSLPE NTAIRTSIAR VSATDADIGT NGEFYYSFKD RTDMFAIHPT
       210        220        230        240        250
SGVIVLTGRL DYLETKLYEM EILAADRGMK LYGSSGISSM AKLTVHIEQA
       260        270        280        290        300
NECAPVITAV TLSPSELDRD PAYAIVTVDD CDQGANGDIA SLSIVAGDLL
       310        320        330        340        350
QQFRTVRSFP GSKEYKVKAI GGIDWDSHPF GYNLTLQAKD KGTPPQFSSV
       360        370        380        390        400
KVIHVTSPQF KAGPVKFEKD VYRAEISEFA PPNTPVVMVK AIPAYSHLRY
       410        420        430        440        450
VFKSTPGKAK FSLNYNTGLI SILEPVKRQQ AAHFELEVTT SDRKASTKVL
       460        470        480        490        500
VKVLGANSNP PEFTQTAYKA AFDENVPIGT TVMSLSAVDP DEGENGYVTY
       510        520        530        540        550
SIANLNHVPF AIDHFTGAVS TSENLDYELM PRVYTLRIRA SDWGLPYRRE
       560        570        580        590        600
VEVLATITLN NLNDNTPLFE KINCEGTIPR DLGVGEQITT VSAIDADELQ
       610        620        630        640        650
LVQYQIEAGN ELDFFSLNPN SGVLSLKRSL MDGLGAKVSF HSLRITATDG
       660        670        680        690        700
ENFATPLYIN ITVAASHKLV NLQCEETGVA KMLAEKLLQA NKLHNQGEVE
       710        720        730        740        750
DIFFDSHSVN AHIPQFRSTL PTGIQVKENQ PVGSSVIFMN STDLDTGFNG
       760        770        780        790        800
KLVYAVSGGN EDSCFMIDME TGMLKILSPL DRETTDKYTL NITVYDLGIP  ← Glycosite 6379 (2) [@N791]
       810        820        830        840        850
QKAAWRLLHV VVVDANDNPP EFLQESYFVE VSEDKEVHSE IIQVEATDKD
       860        870        880        890        900
LGPNGHVTYS IVTDTDTFSI DSVTGVVNIA RPLDRELQHE HSLKIEARDQ
       910        920        930        940        950
AREEPQLFST VVVKVSLEDV NDNPPTFIPP NYRVKVREDL PEGTVIMWLE
       960        970        980        990       1000
AHDPDLGQSG QVRYSLLDHG EGNFDVDKLS GAVRIVQQLD FEKKQVYNLT  ← Glycosite 4491 (5) [@N998]
      1010       1020       1030       1040       1050
VRAKDKGKPV SLSSTCYVEV EVVDVNENLH PPVFSSFVEK GTVKEDAPVG
      1060       1070       1080       1090       1100
SLVMTVSAHD EDARRDGEIR YSIRDGSGVG VFKIGEETGV IETSDRLDRE
      1110       1120       1130       1140       1150
STSHYWLTVF ATDQGVVPLS SFIEIYIEVE DVNDNAPQTS EPVYYPEIME
      1160       1170       1180       1190       1200
NSPKDVSVVQ IEAFDPDSSS NDKLMYKITS GNPQGFFSIH PKTGLITTTS
      1210       1220       1230       1240       1250
RKLDREQQDE HILEVTVTDN GSPPKSTIAR VIVKILDEND NKPQFLQKFY
      1260       1270       1280       1290       1300
KIRLPEREKP DRERNARREP LYHVIATDKD EGPNAEISYS IEDGNEHGKF
      1310       1320       1330       1340       1350
FIEPKTGVVS SKRFSAAGEY DILSIKAVDN GRPQKSSTTR LHIEWISKPK
      1360       1370       1380       1390       1400
PSLEPISFEE SFFTFTVMES DPVAHMIGVI SVEPPGIPLW FDITGGNYDS
      1410       1420       1430       1440       1450
HFDVDKGTGT IIVAKPLDAE QKSNYNLTVE ATDGTTTILT QVFIKVIDTN
      1460       1470       1480       1490       1500
DHRPQFSTSK YEVVIPEDTA PETEILQISA VDQDEKNKLI YTLQSSRDPL
      1510       1520       1530       1540       1550
SLKKFRLDPA TGSLYTSEKL DHEAVHQHTL TVMVRDQDVP VKRNFARIVV
      1560       1570       1580       1590       1600
NVSDTNDHAP WFTASSYKGR VYESAAVGSV VLQVTALDKD KGKNAEVLYS
      1610       1620       1630       1640       1650
IESGNIGNSF MIDPVLGSIK TAKELDRSNQ AEYDLMVKAT DKGSPPMSEI
      1660       1670       1680       1690       1700
TSVRIFVTIA DNASPKFTSK EYSVELSETV SIGSFVGMVT AHSQSSVVYE
      1710       1720       1730       1740       1750
IKDGNTGDAF DINPHSGTII TQKALDFETL PIYTLIIQGT NMAGLSTNTT
      1760       1770       1780       1790       1800
VLVHLQDEND NAPVFMQAEY TGLISESASI NSVVLTDRNV PLVIRAADAD
      1810       1820       1830       1840       1850
KDSNALLVYH IVEPSVHTYF AIDSSTGAIH TVLSLDYEET SIFHFTVQVH
      1860       1870       1880       1890       1900
DMGTPRLFAE YAANVTVHVI DINDCPPVFA KPLYEASLLL PTYKGVKVIT
      1910       1920       1930       1940       1950
VNATDADSSA FSQLIYSITE GNIGEKFSMD YKTGALTVQN TTQLRSRYEL  ← Glycosite 5142 (4) [@N1940]
      1960       1970       1980       1990       2000
TVRASDGRFA GLTSVKINVK ESKESHLKFT QDVYSAVVKE NSTEAETLAV
      2010       2020       2030       2040       2050
ITAIGNPINE PLFYHILNPD RRFKISRTSG VLSTTGTPFD REQQEAFDVV
      2060       2070       2080       2090       2100
VEVTEEHKPS AVAHVVVKVI VEDQNDNAPV FVNLPYYAVV KVDTEVGHVI
      2110       2120       2130       2140       2150
RYVTAVDRDS GRNGEVHYYL KEHHEHFQIG PLGEISLKKQ FELDTLNKEY
      2160       2170       2180       2190       2200
LVTVVAKDGG NPAFSAEVIV PITVMNKAMP VFEKPFYSAE IAESIQVHSP
      2210       2220       2230       2240       2250
VVHVQANSPE GLKVFYSITD GDPFSQFTIN FNTGVINVIA PLDFEAHPAY
      2260       2270       2280       2290       2300
KLSIRATDSL TGAHAEVFVD IIVDDINDNP PVFAQQSYAV TLSEASVIGT
      2310       2320       2330       2340       2350
SVVQVRATDS DSEPNRGISY QMFGNHSKSH DHFHVDSSTG LISLLRTLDY
      2360       2370       2380       2390       2400
EQSRQHTIFV RAVDGGMPTL SSDVIVTVDV TDLNDNPPLF EQQIYEARIS
      2410       2420       2430       2440       2450
EHAPHGHFVT CVKAYDADSS DIDKLQYSIL SGNDHKHFVI DSATGIITLS
      2460       2470       2480       2490       2500
NLHRHALKPF YSLNLSVSDG VFRSSTQVHV TVIGGNLHSP AFLQNEYEVE
      2510       2520       2530       2540       2550
LAENAPLHTL VMEVKTTDGD SGIYGHVTYH IVNDFAKDRF YINERGQIFT
      2560       2570       2580       2590       2600
LEKLDRETPA EKVISVRLMA KDAGGKVAFC TVNVILTDDN DNAPQFRATK
      2610       2620       2630       2640       2650
YEVNIGSSAA KGTSVVKVLA SDADEGSNAD ITYAIEADSE SVKENLEINK
      2660       2670       2680       2690       2700
LSGVITTKES LIGLENEFFT FFVRAVDNGS PSKESVVLVY VKILPPEMQL
      2710       2720       2730       2740       2750
PKFSEPFYTF TVSEDVPIGT EIDLIRAEHS GTVLYSLVKG NTPESNRDES
      2760       2770       2780       2790       2800
FVIDRQSGRL KLEKSLDHET TKWYQFSILA RCTQDDHEMV ASVDVSIQVK
      2810       2820       2830       2840       2850
DANDNSPVFE SSPYEAFIVE NLPGGSRVIQ IRASDADSGT NGQVMYSLDQ
      2860       2870       2880       2890       2900
SQSVEVIESF AINMETGWIT TLKELDHEKR DNYQIKVVAS DHGEKIQLSS
      2910       2920       2930       2940       2950
TAIVDVTVTD VNDSPPRFTA EIYKGTVSED DPQGGVIAIL STTDADSEEI
      2960       2970       2980       2990       3000
NRQVTYFITG GDPLGQFAVE TIQNEWKVYV KKPLDREKRD NYLLTITATD
      3010       3020       3030       3040       3050
GTFSSKAIVE VKVLDANDNS PVCEKTLYSD TIPEDVLPGK LIMQISATDA
      3060       3070       3080       3090       3100
DIRSNAEITY TLLGSGAEKF KLNPDTGELK TSTPLDREEQ AVYHLLVRAT
     3110        3120       3130       3140       3150
DGGGRFCQAS IVLTLEDVND NAPEFSADPY AITVFENTEP GTLLTRVQAT
      3160       3170       3180       3190       3200
DADAGLNRKI LYSLIDSADG QFSINELSGI IQLEKPLDRE LQAVYTLSLK
      3210       3220       3230       3240       3250
AVDQGLPRRL TATGTVIVSV LDINDNPPVF EYREYGATVS EDILVGTEVL
      3260       3270       3280       3290       3300
QVYAASRDIE ANAEITYSII SGNEHGKFSI DSKTGAVFII ENLDYESSHE
      3310       3320       3330       3340       3350
YYLTVEATDG GTPSLSDVAT VNVNVTDIND NTPVFSQDTY TTVISEDAVL
      3360       3370       3380       3390       3400
EQSVITVMAD DADGPSNSHI HYSIIDGNQG SSFTIDPVRG EVKVTKLLDR
      3410       3420       3430       3440       3450
ETISGYTLTV QASDNGSPPR VNTTTVNIDV SDVNDNAPVF SRGNYSVIIQ  ← Glycosite 5857 (7) [@N3422]
      3460       3470       3480       3490       3500
ENKPVGFSVL QLVVTDEDSS HNGPPFFFTI VTGNDEKAFE VNPQGVLLTS
      3510       3520       3530       3540       3550
SAIKRKEKDH YLLQVKVADN GKPQLSSLTY IDIRVIEESI YPPAILPLEI
      3560       3570       3580       3590       3600
FITSSGEEYS GGVIGKIHAT DQDVYDTLTY SLDPQMDNLF SVSSTGGKLI
      3610      3620        3630       3640        3650  ← Glycosite 2933 (3) [@N3613]
AHKKLDIGQY LLNVSVTDGK FTTVADITVH IRQVTQEMLN HTIAIRFANL
      3660       3670       3680       3690       3700  ← Glycosite 4485 (1) [@N3640]
TPEEFVGDYW RNFQRALRNI LGVRRNDIQI VSLQSSEPHP HLDVLLFVEK
      3710       3720       3730       3740       3750
PGSAQISTKQ LLHKINSSVT DIEEIIGVRI LNVFQKLCAG LDCPWKFCDE  ← Glycosite 2557 (6) [@N3716]
      3760       3770       3780       3790       3800
KVSVDESVMS THSTARLSFV TPRHHRAAVC LCKEGRCPPV HHGCEDDPCP
      3810       3820       3830       3840       3850
EGSECVSDPW EEKHTCVCPS GRFGQCPGSS SMTLTGNSYV KYRLTENENK
      3860       3870       3880       3890       3900
LEMKLTMRLR TYSTHAVVMY ARGTDYSILE IHHGRLQYKF DCGSGPGIVS
      3910       3920       3930       3940       3950
VQSIQVNDGQ WHAVALEVNG NYARLVLDQV HTASGTAPGT LKTLNLDNYV
      3960       3970       3980       3990       4000
FFGGHIRQQG TRHGRSPQVG NGFRGCMDSI YLNGQELPLN SKPRSYAHIE
      4010       4020       4030       4040       4050
ESVDVSPGCF LTATEDCASN PCQNGGVCNP SPAGGYYCKC SALYIGTHCE
      4060         4070       4080      4090         4100
ISVNPCSSKP CLYGGTCVVD NGGFVCQCRG LYTGQRCQLS PYCKDEPCKN
      4110       4120       4130       4140       4150
GGTCFDSLDG AVCQCDSGFR GERCQSDIDE CSGNPCLHGA LCENTHGSYH
      4160       4170       4180       4190       4200
CNCSHEYRGR HCEDAAPNQY VSTPWNIGLA EGIGIVVFVA GIFLLVVVFV
      4210       4220       4230       4240       4250
LCRKMISRKK KHQAEPKDKH LGPATAFLQR PYFDSKLNKN IYSDIPPQVP
      4260       4270       4280       4290       4300
VRPISYTPSI PSDSRNNLDR NSFEGSAIPE HPEFSTFNPE SVHGHRKAVA
      4310       4320       4330       4340       4350
VCSVAPNLPP PPPSNSPSDS DSIQKPSWDF DYDTKVVDLD PCLSKKPLEE
      4360       4370       4380       4390       4400
KPSQPYSARE SLSEVQSLSS FQSESCDDNG YHWDTSDWMP SVPLPDIQEF
      4410       4420       4430       4440       4450
PNYEVIDEQT PLYSADPNAI DTDYYPGGYD IESDFPPPPE DFPAADELPP
      4460       4470       4480       4490       4500
LPPEFSNQFE SIHPPRDMPA AGSLGSSSRN RQRFNLNQYL PNFYPLDMSE
      4510       4520       4530       4540       4550
PQTKGTGENS TCREPHAPYP PGYQRHFEAP AVESMPMSVY ASTASCSDVS
      4560       4570       4580
ACCEVESEVM MSDYESGDDG HFEEVTIPPL DSQQHTEV
```

L1CAM (Neural cell adhesion molecule L1)

Data ID[a] (Glycosite #)[b]	Peptide sequence	Ratio of sialoglycosite abundance in 1,3,4-O-Bu_3ManNAc-treated to control cells: MCF10A	T-47D	MDA-MB-231
**715[a] (1)[b]**	DHVVVPA**NTT**SVILSGLR	5.48	2.54	1.51
**1440 (2)**	FFPYA**NGT**LGIR P32004	1.30	2.29	1.36
**1748 (3)**	GEG**NET**TNMVITWKPLR	0.22	2.05	1.14
**2505 (4)**	LTAD**NQT**YMAVQGSTAYLLCK	10.37	2.09	1.14
**5178 (5)**	H**NLT**DLSPHLR	0.93	2.24	1.12
**3238 (6)**	LLFPT**NSS**SHLVALQGQPLVLECIAEGFPTPTIK	0.00	2.22	0.88
**1041 (7)**	EELGVTVYQSPHSGSFTITG**NNS**NFAQR	7.04	0.42	0.86
**2089 (8)**	GY**NVT**YWR	1.07	2.07	0.84
**1985 (9)**	GSGPASEFTFSTPEGVPGHPEALHLECQS**NTS**LLLR	0.03	1.18	0.74
**3633 (10)**	LVIHSLDYSDQG**NYS**CVASTELDVVESR	2.00	2.29	0.05

[a] The data ID number is provided in **Column A** of **Sheet 1** of **Supplemental File #3**

[b] The glycosite # represents the labeling scheme used in **Figure 4** of the main text

Amino acid sequence (*from* Uniprot; https://www.uniprot.org/uniprot/P32004)

N-glycan consensus sequons in **red** are predicted by NetNGly to be occupied

N-glycan consensus sequons in **blue** are predicted by NetNGly to be NOT occupied

Sequences underlined using a dotted line represent peptide sequences identified as “glycosites”

```
        10         20         30         40         50
MVVALRYVWP LLLCSPCLLI QIPEEYEGHH VMEPPVITEQ SPRRLVVFPT
        60         70         80         90        100
DDISLKCEAS GKPEVQFRWT RDGVHFKPKE ELGVTVYQSP HSGSFTITGN  ← Glycosite 1041 (7) [@N100]
       110        120        130        140        150
NSNFAQRFQG IYRCFASNKL GTAMSHEIRL MAEGAPKWPK ETVKPVEVEE
       160        170        180        190        200
GESVVLPCNP PPSAEPLRIY WMNSKILHIK QDERVTMGQN GNLYFANVLT
       210        220        230        240        250
SDNHSDYICH AHFPGTRTII QKEPIDLRVK ATNSMIDRKP RLLFPTNSSS  ← Glycosite 3238 (6) [@N247]
       260        270        280        290        300
HLVALQGQPL VLECIAEGFP TPTIKWLRPS GPMPADRVTY QNHNKTLQLL
       310        320        330        340        350
KVGEEDDGEY RCLAENSLGS ARHAYYVTVE AAPYWLHKPQ SHLYGPGETA
       360        370        380        390        400
RLDCQVQGRP QPEVTWRING IPVEELAKDQ KYRIQRGALI LSNVQPSDTM
       410        420        430        440        450
VTQCEARNRH GLLLANAYIY VVQLPAKILT ADNQTYMAVQ GSTAYLLCKA  ← Glycosite 2504 (4) [@N433]
       460        470        480        490        500
FGAPVPSVQW LDEDGTTVLQ DERFFPYANG TLGIRDLQAN DTGRYFCLAA  ← Glycosite 1440 (2) [@N479]
       510        520        530        540        550
NDQNNVTIMA NLKVKDATQI TQGPRSTIEK KGSRVTFTCQ ASFDPSLQPS
       560        570        580        590        600
ITWRGDGRDL QELGDSDKYF IEDGRLVIHS LDYSDQGNYS CVASTELDVV  ← Glycosite 3663 (10) [@N588]
       610        620        630        640        650
ESRAQLLVVG SPGPVPRLVL SDLHLLTQSQ VRVSWSPAED HNAPIEKYDI
       660        670        680        690        700
EFEDKEMAPE KWYSLGKVPG NQTSTTLKLS PYVHYTFRVT AINKYGPGEP
       710        720        730        740        750
SPVSETVVTP EAAPEKNPVD VKGEGNETTN MVITWKPLRW MDWNAPQVQY  ← Glycosite 1748 (3) [@N726]
       760        770        780        790        800
RVQWRPQGTR GPWQEQIVSD PFLVVSNTST FVPYEIKVQA VNSQGKGPEP
       810        820        830        840        850
QVTIGYSGED YPQAIPELEG IEILNSSAVL VKWRPVDLAQ VKGHLRGYNV  ← Glycosite 2089 (8) [@N849]
       860        870        880        890        900
TYWREGSQRK HSKRHIHKDH VVVPANTTSV ILSGLRPYSS YHLEVQAFNG  ← Glycosite 715 (1) [@N876]
       910        920        930        940        950
RGSGPASEFT FSTPEGVPGH PEALHLECQS NTSLLLRWQP PLSHNGVLTG  ← Glycosite 1985 (9) [@N931]
       960        970        980        990       1000
YVLSYHPLDE GGKGQLSFNL RDPELRTHNL TDLSPHLRYR FQLQATTKEG  ← Glycosite 5178 (5) [@971]
      1010       1020       1030       1040       1050
PGEAIVREGG TMALSGISDF GNISATAGEN YSVVSWVPKE GQCNFRFHIL
      1060       1070       1080       1090       1100
FKALGEEKGG ASLSPQYVSY NQSSYTQWDL QPDTDYEIHL FKERMFRHQM
      1110       1120       1130       1140       1150
AVKTNGTGRV RLPPAGFATE GWFIGFVSAI ILLLLVLLIL CFIKRSKGGK
      1160       1170       1180       1190       1200
YSVKDKEDTQ VDSEARPMKD ETFGEYRSLE SDNEEKAFGS SQPSLNGDIK
      1210       1220       1230       1240       1250
PLGSDDSLAD YGGSVDVQFN EDGSFIGQYS GKKEKEAAGG NDSSGATSPI

NPAVALE
```

TENM3 (Teneurin-3)

Data ID[a] (Glycosite #)[b]	Peptide sequence	Ratio of sialoglycosite abundance in 1,3,4-O-Bu_3ManNAc-treated to control cells: MCF10A	T-47D	MDA-MB-231
3074[a] (1[b])	LGGFTQE**NNT**IDSGELDIGRR	1.24	7.06	2.50
4567 (2)	R**NMT**LPGENGQNLVEWR	0.38	13.76	2.02
1558 (3)	F**NIS**LQK	0.65	32.93	1.60
1971 (4)	GQVLTADGTPLIGV**NVS**FFHYPEYGYTITR	1.44	40.79	1.42
4105 (5)	**NMT**LPGENGQNLVEWR	2.53	3.03	0.57
2370 (6)	IGPFA**NTT**K	1.01	2.00	0.57
3329 (7)	LMAV**NVT**YSSTGQIASIQR	1.24	2.00	0.52
3073 (8)	LGGFTQE**NNT**IDSGELDIGR	0.90	2.28	0.48
3581 (9)	LT**NVT**FPTGVVTNLHGDMDK	0.72	2.35	0.29 2

[a] The data ID number is provided in **Column A** of **Sheet 1** of **Supplemental File #3**

[b] The glycosite # represents the labeling scheme used in **Figure 4** of the main text

Amino acid sequence (*from* Uniprot; https://www.uniprot.org/uniprot/Q92673)

N-glycan consensus sequons in **red** are predicted by NetNGly to be occupied

N-glycan consensus sequons in **blue** are predicted by NetNGly to be NOT occupied

Sequences underlined using a dotted line represent peptide sequences identified as "glycosites"

```
        10         20         30         40         50
MDVKERRPYC SLTKSRREKE RRYTNSSADN EECRVPTQKS YSSSETLKAF
        60         70         80         90        100
DHDSSRLLYG NRVKDLVHRE ADEFTRQGQN FTLRQLGVCE PATRRGLAFC
       110        120        130        140        150
AEMGLPHRGY SISAGSDADT ENEAVMSPEH AMRLWGRGVK SGRSSCLSSR
       160        170        180        190        200
SNSALTLTDT EHENKSDSEN EQPASNQGQS TLQPLPPSHK QHSAQHHPSI
       210        220        230        240        250
TSLNRNSLTN RRNQSPAPPA ALPAELQTTP ESVQLQDSWV LGSNVPLESR
       260        270        280        290        300
HFLFKTGTGT TPLFSTATPG YTMASGSVYS PPTRPLPRNT LSRSAFKFKK
       310        320        330        340        350
SSKYCSWKCT ALCAVGVSVL LAILLSYFIA MHLFGLNWQL QQTENDTFEN
       360        370        380        390        400
GKVNSDTMPT NTVSLPSGDN GKLGGFTQEN NTIDSGELDI GRRAIQEIPP  ← Glycosite 3074 (1) [@N380]
       410        420        430        440        450
GIFWRSQLFI DQPQFLKFNI SLQKDALIGV YGRKGLPPSH TQYDFVELLD  ← Glycosite 1558 (3) [@N419]
       460        470        480        490        500
GSRLIAREQR SLLETERAGR QARSVSLHEA GFIQYLDSGI WHLAFYNDGK
       510        520        530        540        550
NAEQVSFNTI VIESVVECPR NCHGNGECVS GTCHCFPGFL GPDCSRAACP
       560        570        580        590        600
VLCSGNGQYS KGRCLCFSGW KGTECDVPTT QCIDPQCGGR GICIMGSCAC
       610        620        630        640        650
NSGYKGESCE EADCIDPGCS NHGVCIHGEC HCSPGWGGSN CEILKTMCPD
       660        670        680        690        700
QCSGHGTYLQ ESGSCTCDPN WTGPDCSNEI CSVDCGSHGV CMGGTCRCEE
       710        720        730        740        750
GWTGPACNQR ACHPRCAEHG TCKDGKCECS QGWNGEHCTI EGCPGLCNSN
       760        770        780        790        800
GRCTLDQNGW HCVCQPGWRG AGCDVAMETL CTDSKDNEGD GLIDCMDPDC
       810        820        830        840        850
CLQSSCQNQP YCRGLPDPQD IISQSLQSPS QQAAKSFYDR ISFLIGSDST
       860        870        880        890        900
HVIPGESPFN KSLASVIRGQ VLTADGTPLI GVNVSFFHYP EYGYTITRQD  ← Glycosite 1971 (4) [@N883]
       910        920        930        940        950
GMFDLVANGG ASLTLVFERS PFLTQYHTVW IPWNVFYVMD TLVMKKEEND
       960        970        980        990       1000
IPSCDLSGFV RPNPIIVSSP LSTFFRSSPE DSPIIPETQV LHEETTIPGT
      1010       1020       1030       1040       1050
DLKLSYLSSR AAGYKSVLKI TMTQSIIPFN LMKVHLMVAV VGRLFQKWFP
      1060       1070       1080       1090       1100
ASPNLAYTFI WDKTDAYNQK VYGLSEAVVS VGYEYESCLD LTLWEKRTAI
      1110       1120       1130       1140       1150
LQGYELDASN MGGWTLDKHH VLDVQNGILY KGNGENQFIS QQPPVVSSIM
      1160       1170       1180       1190       1200
GNGRRRSISC PSCNGQADGN KLLAPVALAC GIDGSLYVGD FNYVRRIFPS
      1210       1220       1230       1240       1250
GNVTSVLELS SNPAHRYYLA TDPVTGDLYV SDTNTRRIYR PKSLTGAKDL
      1260       1270       1280       1290       1300
TKNAEVVAGT GEQCLPFDEA RCGDGGKAVE ATLMSPKGMA VDKNGLIYFV
      1310       1320       1330       1340       1350
DGTMIRKVDQ NGIISTLLGS NDLTSARPLT CDTSMHISQV RLEWPTDLAI  ← Glycosite 4612 (1) [@N1164]
      1360       1370       1380       1390       1400
NPMDNSIYVL DNNVVLQITE NRQVRIAAGR PMHCQVPGVE YPVGKHAVQT
      1410       1420       1430       1440       1450
TLESATAIAV SYSGVLYITE TDEKKINRIR QVTTDGEISL VAGIPSECDC
      1460       1470       1480       1490       1500
KNDANCDCYQ SGDGYAKDAK LSAPSSLAAS PDGTLYIADL GNIRIRAVSK
      1510       1520       1530       1540       1550
NKPLLNSMNF YEVASPTDQE LYIFDINGTH QYTVSLVTGD YLYNFSYSND
      1560       1570       1580       1590       1600
NDITAVTDSN GNTLRIRRDP NRMPVRVVSP DNQVIWLTIG TNGCLKSMTA
      1610       1620       1630       1640       1650
QGLELVLFTY HGNSGLLATK SDETGWTTFF DYDSEGRLTN VTFPTGVVTN  ← Glycosite 3581 (9) [@N1640]
      1660       1670       1680       1690       1700
LHGDMDKAIT VDIESSSREE DVSITSNLSS IDSFYTMVQD QLRNSYQIGY
      1710       1720       1730       1740       1750
DGSLRIIYAS GLDSHYQTEP HVLAGTANPT VAKRNMTLPG ENGQNLVEWR  ← Glycosite 4567 (2) [@N1735]
      1760       1770       1780       1790       1800
FRKEQAQGKV NVFGRKLRVN GRNLLSVDFD RTTKTEKIYD DHRKFLLRIA
      1810       1820       1830       1840       1850
YDTSGHPTLW LPSSKLMAVN VTYSSTGQIA SIQRGTTSEK VDYDGQGRIV
      1860       1870       1880       1890       1900
SRVFADGKTW SYTYLEKSMV LLLHSQRQYI FEYDMWDRLS AITMPSVARH
      1910       1920       1930       1940       1950
TMQTIRSIGY YRNIYNPPES NASIITDYNE EGLLLQTAFL GTSRRVLFKY
      1960       1970       1980       1990       2000
RRQTRLSEIL YDSTRVSFTY DETAGVLKTV NLQSDGFICT IRYRQIGPLI
      2010       2020       2030       2040       2050
DRQIFRFSED GMVNARFDYS YDNSFRVTSM QGVINETPLP IDLYQFDDIS
      2060       2070       2080       2090       2100
GKVEQFGKFG VIYYDINQII STAVMTYTKH FDAHGRIKEI QYEIFRSLMY
      2110       2120       2130       2140       2150
WITIQYDNMG RVTKREIKIG PFANTTKYAY EYDVDGQLQT VYLNEKIMWR  ← Glycosite 2370 (6) [@N2124]
      2160       2170       2180       2190       2200
YNYDLNGNLH LLNPSNSARL TPLRYDLRDR ITRLGDVQYR LDEDGFLRQR
      2210       2220       2230       2240       2250
GTEIFEYSSK GLLTRVYSKG SGWTVIYRYD GLGRRVSSKT SLGQHLQFFY
      2260       2270       2280       2290       2300
ADLTYPTRIT HVYNHSSSEI TSLYYDLQGH LFAMEISSGD EFYIASDNTG
      2310       2320       2330       2340       2350
TPLAVFSSNG LMLKQIQYTA YGEIYFDSNI DFQLVIGFHG GLYDPLTKLI
      2360       2370       2380       2390       2400
HFGERDYDIL AGRWTTPDIE IWKRIGKDPA PFNLYMFRNN NPASKIHDVK
      2410       2420       2430       2440       2450
DYITDVNSWL VTFGFHLHNA IPGFPVPKFD LTEPSYELVK SQQWDDIPPI
      2460       2470       2480       2490       2500
FGVQQQVARQ AKAFLSLGKM AEVQVSRRRA GGAQSWLWFA TVKSLIGKGV
      2510       2520       2530       2540       2550
MLAVSQGRVQ TNVLNIANED CIKVAAVLNN AFYLENLHFT IEGKDTHYFI
      2560       2570       2580       2590       2600
KTTTPESDLG TLRLTSGRKA LENGINVTVS QSTTVVNGRT RRFADVEMQF
      2610       2620       2630       2640       2650
GALALHVRYG MTLDEEKARI LEQARQRALA RAWAREQQRV RDGEEGARLW
      2660       2670       2680       2690
TEGEKRQLLS AGKVQGYDGY YVLSVEQYPE LADSANNIQF LRQSEIGRR
```

PTPRJ (Receptor-type tyrosine-protein phosphatase eta)

Data ID[a] (Glycosite #)[b]	Peptide sequence	Ratio of sialoglycosite abundance in 1,3,4-O-Bu_3ManNAc-treated to control cells: MCF10A	T-47D	MDA-MB-231
**6309[a] (1[b])**	Y**NAT**VYSQAA**NGT**EGQPQAIEFR	2.22	2.30	1.43
**3475 (2)**	LQV**NIS**GLK	2.36	1.29	1.29
**6170 (3)**	YEIDVG**NES**TTLGYYNGK	18.05	1.71	1.14
**52 (4)**	ACVAGFT**NIT**FHPQNK	32.30	1.95	1.11
**5555 (5)**	VEITT**NQS**IIIGGLFPGTK	0.03	28.07	0.16

[a] The data ID number is provided in **Column A** of **Sheet 1** of **Supplemental File #3**

[b] The glycosite # represents the labeling scheme used in **Figure 4** of the main text

Amino acid sequence (*from* Uniprot; https://www.uniprot.org/uniprot/Q12913)

N-glycan consensus sequons in **red** are predicted by NetNGly to be occupied

N-glycan consensus sequons in **blue** are predicted by NetNGly to be NOT occupied

Sequences underlined using a dotted line represent peptide sequences identified as “glycosites”

```
        10         20         30         40         50
MKPAAREARL PPRSPGLRWA LPLLLLLLRL GQILCAGGTP SPIPDPSVAT
        60         70         80         90        100
VATGENGITQ ISSTAESFHK QNGTGTPQVE TNTSEDGESS GANDSLRTPE
       110        120        130        140        150
QGSNGTDGAS QKTPSSTGPS PVFDIKAVSI SPTNVILTWK SNDTAASEYK
       160        170        180        190        200
YVVKHKMENE KTITVVHQPW CNITGLRPAT SYVFSITPGI GNETWGDPRV
       210        220        230        240        250
IKVITEPIPV SDLRVALTGV RKAALSWSNG NGTASCRVLL ESIGSHEELT
       260        270        280        290        300
QDSRLQVNIS GLKPGVQYNI NPYLLQSNKT KGDPLGTEGG LDASNTERSR
       310        320        330        340        350
AGSPTAPVHD ESLVGPVDPS SGQQSRDTEV LLVGLEPGTR YNATVYSQAA
       360        370        380        390        400
NGTEGQPQAI EFRTNAIQVF DVTAVNISAT SLTLIWKVSD NESSSNYTYK
       410        420        430        440        450
IHVAGETDSS NLNVSEPRAV IPGLRSSTFY NITVCPVLGD IEGTPGFLQV
       460        470        480        490        500
HTPPVPVSDF RVTVVSTTEI GLAWSSHDAE SFQMHITQEG AGNSRVEITT
       510        520        530        540        550
NQSIIIGGLF PGTKYCFEIV PKGPNGTEGA SRTVCNRTVP SAVFDIHVVY
       560        570        580        590        600
VTTTEMWLDW KSPDGASEYV YHLVIESKHG SNHTSTYDKA ITLQGLIPGT
       610        620        630        640        650
LYNITISPEV DHVWGDPNST AQYTRPSNVS NIDVSTNTTA ATLSWQNFDD
       660        670        680        690        700
ASPTYSYCLL IEKAGNSS NA TQVVTDIGIT DATVTELIPG SSYTVEIFAQ
       710        720        730        740        750
VGDGIKSLEP GRKSFCTDPA SMASFDCEVV PKEPALVLKW TCPPGANAGF
       760        770        780        790        800
ELEVSSGAWN NATHLESCSS ENGTEYRTEV TYLNFSTSYN ISITTVSCGK
       810        820        830        840        850
MAAPTRNTCT TGITDPPPPD GSPNITSVSH NSVKVKFSGF EASHGPIKAY
       860        870        880        890        900
AVILTTGEAG HPSADVLKYT YEDFKKGASD TYVTYLIRTE EKGRSQSLSE
       910        920        930        940        950
VLKYEIDVGN ESTTLGYYNG KLEPLGSYRA CVAGFTNITF HPQNKGLIDG
       960        970        980        990       1000
AESYVSFSRY SDAVSLPQDP GVICGAVFGC IFGALVIVTV GGFIFWRKKR
      1010       1020       1030       1040       1050
KDAKNNEVSF SQIKPKKSKL IRVENFEAYF KKQQADSNCG FAEEYEDLKL
      1060       1070       1080       1090       1100
VGISQPKYAA ELAENRGKNR YNNVLPYDIS RVKLSVQTHS TDDYINANYM
      1110       1120       1130       1140       1150
PGYHSKKDFI ATQGPLPNTL KDFWRMVWEK NVYAIIMLTK CVEQGRTKCE
      1160       1170       1180       1190       1200
EYWPSKQAQD YGDITVAMTS EIVLPEWTIR DFTVKNIQTS ESHPLRQFHF
      1210       1220       1230       1240       1250
TSWPDHGVPD TTDLLINFRY LVRDYMKQSP PESPILVHCS AGVGRTGTFI
      1260       1270       1280       1290       1300
AIDRLIYQIE NENTVDVYGI VYDLRMHRPL MVQTEDQYVF LNQCVLDIVR
      1310       1320       1330
SQKDSKVDLI YQNTTAMTIY ENLAPVTTFG KTNGYIA
```

← **Glycosite 3475 (2)** [@N258]

← **Glycosite 6309 (1)** [@N342 or N361]

(note: this peptides contains two possible sites of N-glycosylation)

← **Glycosite 5555 (5)** [@N501]

Glycosite 6170 (3) [@N910]

Glycosite 52 (4) [@N937]

SORL1 (Sortilin-related receptor)

Data ID[a] (Glycosite #)[b]	Peptide sequence	Ratio of sialoglycosite abundance in 1,3,4-O-Bu_3ManNAc-treated to control cells: MCF10A	T-47D	MDA-MB-231
**4642[a] (1[b])**	SDEY**NCS**SGMCIR	2.48	1.46	1.87
**207 (2)**	AI**NQT**AVECTWTGPR	2.06	0.18	1.33
**5633 (3)**	VG**NLT**AHTSYEISAWAK	4.49	77.19	1.27
**3565 (4)**	LTIV**NSS**VLDRPR	1.04	1.95	1.00
**33 (5)**	AAS**NFT**EIK	4.93	0.37	0.99
**6066 (6)**	VYGQVSL**NDS**HNQMVVHWAGEK	1.40	2.99	0.73
**2079 (7)**	GYEIHMFDSAM**NIT**AYLG**NTT**DNFFK	2.35	2.42	0.73
**6221 (8)**	YHIIVQLG**NMS**K	2.05	2.41	0.67
**5552 (9)**	VEGLQGVYIATLI**NGS**MNEENMR	1.67	272.6	0.38
**3761 (10)**	MGH**NYT**FTVQAR	0.67	2.44	0.22

[a] The data ID number is provided in Column A of Sheet 1 of the **Supp_Mat_sialoglycosite_raw_data.xls** file

[b] The glycosite # represents the labeling scheme used in **Figure 4** of the main text

Amino acid sequence (*from* Uniprot; https://www.uniprot.org/uniprot/Q92673)

N-glycan consensus sequons in **red** are predicted by NetNGly to be occupied

N-glycan consensus sequons in **blue** are predicted by NetNGly to be NOT occupied

Sequences underlined using a dotted line represent peptide sequences identified as "glycosites"

```
        10         20         30         40         50
MATRSSRRES RLPFLFTLVA LLPPGALCEV WTQRLHGGSA PLPQDRGFLV
        60         70         80         90        100
VQGDPRELRL WARGDARGAS RADEKPLRRK RSAALQPEPI KVYGQVSLND   ← Glycosite 6066 (6) [@N99]
       110        120        130        140        150
SHNQMVVHWA GEKSNVIVAL ARDSLALARP KSSDVYVSYD YGKSFKKISD
       160        170        180        190        200
KLNFGLGNRS EAVIAQFYHS PADNKRYIFA DAYAQYLWIT FDFCNTLQGF
       210        220        230        240        250
SIPFRAADLL LHSKASNLLL GFDRSHPNKQ LWKSDDFGQT WIMIQEHVKS
       260        270        280        290        300
FSWGIDPYDK PNTIYIERHE PSGYSTVFRS TDFFQSRENQ EVILEEVRDF
       310        320        330        340        350
QLRDKYMFAT KVVHLLGSEQ QSSVQLWVSF GRKPMRAAQF VTRHPINEYY
       360        370        380        390        400
IADASEDQVF VCVSHSNNRT NLYISEAEGL KFSLSLENVL YYSPGGAGSD
       410        420        430        440        450
TLVRYFANEP FADFHRVEGL QGVYIATLIN GSMNEENMRS VITFDKGGTW   ← Glycosite 5552 (9) [@N430]
       460        470        480        490        500
EFLQAPAFTG YGEKINCELS QGCSLHLAQR LSQLLNLQLR RMPILSKESA
       510        520        530        540        550
PGLIIATGSV GKNLASKTNV YISSSAGARW REALPGPHYY TWGDHGGIIT
       560        570        580        590        600
AIAQGMETNE LKYSTNEGET WKTFIFSEKP VFVYGLLTEP GEKSTVFTIF
       610        620        630        640        650
GSNKENVHSW LILQVNATDA LGVPCTENDY KLWSPSDERG NECLLGHKTV
       660        670        680        690        700
FKRRTPHATC FNGEDFDRPV VVSNCSCTRE DYECDFGFKM SEDLSLEVCV
       710        720        730        740        750
PDPEFSGKSY SPPVPCPVGS TYRRTRGYRK ISGDTCSGGD VEARLEGELV
       760        770        780        790        800
PCPLAEENEF ILYAVRKSIY RYDLASGATE QLPLTGLRAA VALDFDYEHN
       810        820        830        840        850
CLYWSDLALD VIQRLCLNGS TGQEVIINSG LETVEALAFE PLSQLLYWVD
       860        870        880        890        900
AGFKKIEVAN PDGDFRLTIV NSSVLDRPRA LVLVPQEGVM FWTDWGDLKP   ← Glycosite 3564 (4) [@N871]
       910        920        930        940        950
GIYRSNMDGS AAYHLVSEDV KWPNGISVDD QWIYWTDAYL ECIERITFSG
       960        970        980        990       1000
QQRSVILDNL PHPYAIAVFK NEIYWDDWSQ LSIFRASKYS GSQMEILANQ
      1010       1020       1030       1040       1050
LTGLMDMKIF YKGKNTGSNA CVPRPCSLLC LPKANNSRSC RCPEDVSSSV
      1060       1070       1080       1090       1100
LPSGDLMCDC PQGYQLKNNT CVKQENTCLR NQYRCSNGNC INSIWWCDFD
      1110       1120       1130       1140       1150
NDCGDMSDER NCPTTICDLD TQFRCQESGT CIPLSYKCDL EDDCGDNSDE
      1160       1170       1180       1190       1200
SHCEMHQCRS DEYNCSSGMC IRSSWVCDGD NDCRDWSDEA NCTAIYHTCE   ← Glycosite 4612 (1) [@N1164]
      1210       1220       1230       1240       1250
ASNFQCRNGH CIPQRWACDG DTDCQDGSDE DPVNCEKKCN GFRCPNGTCI
      1260       1270       1280       1290       1300
PSSKHCDGLR DCSDGSDEQH CEPLCTHFMD FVCKNRQQCL FHSMVCDGII
      1310       1320       1330       1340       1350
QCRDGSDEDA AFAGCSQDPE FHKVCDEFGF QCQNGVCISL IWKCDGMDDC
      1360       1370       1380       1390       1400
GDYSDEANCE NPTEAPNCSR YFQFRCENGH CIPNRWKCDR ENDCGDWSDE
      1410       1420       1430       1440       1450
KDCGDSHILP FSTPGPSTCL PNYYRCSSGT CVMDTWVCDG YRDCADGSDE
      1460       1470       1480       1490       1500
EACPLLANVT AASTPTQLGR CDRFEFECHQ PKTCIPNWKR CDGHQDCQDG
      1510       1520       1530       1540       1550
RDEANCPTHS TLTCMSREFQ CEDGEACIVL SERCDGFLDC SDESDEKACS
      1560       1570       1580       1590       1600
DELTVYKVQN LQWTADFSGD VTLTWMRPKK MPSASCVYNV YYRVVGESIW
      1610       1620       1630       1640       1650
KTLETHSNKT NTVLKVLKPD TTYQVKVQVQ CLSKAHNTND FVTLRTPEGL
      1660       1670       1680       1690       1700
PDAPRNLQLS LPREAEGVIV GHWAPPIHTH GLIREYIVEY SRSGSKMWAS
      1710       1720       1730       1740       1750
QRAASNFTEI KNLLVNTLYT VRVAAVTSRG IGNWSDSKSI TTIKGKVIPP   ← Glycosite 33 (5) [@N1706]
      1760       1770       1780       1790       1800
PDIHIDSYGE NYLSFTLTME SDIKVNGYVV NLFWAFDTHK QERRTLNFRG
      1810       1820       1830       1840       1850
SILSHKVGNL TAHTSYEISA WAKTDLGDSP LAFEHVMTRG VRPPAPSLKA   ← Glycosite 5633 (3)
      1860       1870       1880       1890       1900
KAINQTAVEC TWTGPRNVVY GIFYATSFLD LYRNPKSLTT SLHNKTVIVS
      1910       1920       1930       1940       1950
KDEQYLFLVR VVVPYQGPSS DYVVVKMIPD SRLPPRHLHV VHTGKTSVVI
      1960       1970       1980       1990       2000
KWESPYDSPD QDLLYAVAVK DLIRKTDRSY KVKSRNSTVE YTLNKLEPGG
      2010       2020       2030       2040       2050
KYHIIVQLGN MSKDSSIKIT TVSLSAPDAL KIITENDHVL LFWKSLALKE   ← Glycosite 6221 (8) @N2010]
      2060       2070       2080       2090       2100
KHFNESRGYE IHMFDSAMNI TAYLGNTTDN FFKISNLKMG HNYTFTVQAR   ← Glycosite 2079 (7) [two sites @N2069 and N2076]
      2110       2120       2130       2140       2150      ← Glycosite 3761 (10) [@N2092]
CLFGNQICGE PAILLYDELG SGADASATQA ARSTDVAAVV VPILFLILLS
      2160       2170       2180       2190       2200
LGVGFAILYT KHRRLQSSFT AFANSHYSSR LGSAIFSSGD DLGEDDEDAP
      2210
MITGFSDDVP MVIA
```

HYOU1 (Hypoxia up-regulated protein)

Data ID[a] (Glycosite #)[b]	Peptide sequence	Ratio of sialoglycosite abundance in 1,3,4-O-Bu_3ManNAc-treated to control cells: MCF10A	T-47D	MDA-MB-231
**5588 (1)**	VFGSQ**NLT**TVK	1.62	2.63	1.29
**5688 (2)**	VI**NET**WAWK	1.59	2.35	1.22
**102 (3)**	AEPPL**NAS**ASDQGEK	1.62	2.28	1.17
**737 (4)**	DI**NTT**AQNIMFYDMGSGSTVCTIVTYQMVK	153.65	3.70	0.02
**1218 (5)**	E**NGT**DTVQEEEESPAEGSK	356.41	1.76	0.003

[a] The data ID number is provided in **Column A** of **Sheet 1** of **Supplemental File #3**

[b] The glycosite # represents the labeling scheme used in **Figure 4** of the main text

Amino acid sequence (*from* Uniprot; https://www.uniprot.org/uniprot/Q9Y4L1)

N-glycan consensus sequons in **red** are predicted by NetNGly to be occupied

N-glycan consensus sequons in **blue** are predicted by NetNGly to be NOT occupied

Sequences underlined using a dotted line represent peptide sequences identified as "glycosites"

```
        10         20         30         40         50
MADKVRRQRP RRRVCWALVA VLLADLLALS DTLAVMSVDL GSESMKVAIV
        60         70         80         90        100
KPGVPMEIVL NKESRRKTPV IVTLKENERF FGDSAASMAI KNPKATLRYF
       110        120        130        140        150
QHLLGKQADN PHVALYQARF PEHELTFDPQ RQTVHFQISS QLQFSPEEVL
       160        170        180        190        200
GMVLNYSRSL AEDFAEQPIK DAVITVPVFF NQAERRAVLQ AARMAGLKVL
       210        220        230        240        250
QLINDNTATA LSYGVFRRKD INTTAQNIMF YDMGSGSTVC TIVTYQMVKT  ← Glycosite 737 (4) [@N222]
       260        270        280        290        300
KEAGMQPQLQ IRGVGFDRTL GGLEMELRLR ERLAGLFNEQ RKGQRAKDVR
       310        320        330        340        350
ENPRAMAKLL REANRLKTVL SANADHMAQI EGLMDDVDFK AKVTRVEFEE
       360        370        380        390        400
LCADLFERVP GPVQQALQSA EMSLDEIEQV ILVGGATRVP RVQEVLLKAV
       410        420        430        440        450
GKEELGKNIN ADEAAAMGAV YQAAALSKAF KVKPFVVRDA VVYPILVEFT
       460        470        480        490        500
REVEEEPGIH SLKHNKRVLF SRMGPYPQRK VITFNRYSHD FNFHINYGDL
       510        520        530        540        550
GFLGPEDLRV FGSQNLTTVK LKGVGDSFKK YPDYESKGIK AHFNLDESGV  ← Glycosite 5588 (1) [@N515]
       560        570        580        590        600
LSLDRVESVF ETLVEDSAEE ESTLTKLGNT ISSLFGGGTT PDAKENGTDT  ← Glycosite 1218 (5) [@N594]
       610        620        630        640        650
VQEEEESPAE GSKDEPGEQV ELKEEAEAPV EDGSQPPPPE PKGDATPEGE
       660        670        680        690        700
KATEKENGDK SEAQKPSEKA EAGPEGVAPA PEGEKKQKPA RKRRMVEEIG
       710        720        730        740        750
VELVVLDLPD LPEDKLAQSV QKLQDLTLRD LEKQEREKAA NSLEAFIFET
       760        770        780        790        800
QDKLYQPEYQ EVSTEEQREE ISGKLSAAST WLEDEGVGAT TVMLKEKLAE
       810        820        830        840        850
LRKLCQGLFF RVEERKKWPE RLSALDNLLN HSSMFLKGAR LIPEMDQIFT
       860        870        880        890        900
EVEMTTLEKV INETWAWKNA TLAEQAKLPA TEKPVLLSKD IEAKMMALDR  ← Glycosite 5688 (2) [@N862]
       910        920        930        940        950
EVQYLLNKAK FTKPRPRPKD KNGTRAEPPL NASASDQGEK VIPPAGQTED  ← Glycosite 102 (3) [@N931]
       960        970        980        990
AEPISEPEKV ETGSEPGDTE PLELGGPGAE PEQKEQSTGQ KRPLKNDEL
```

FKBP10 (Peptidyl-prolyl cis-trans isomerase FKBP10)

Data ID[a] (Glycosite #)[b]	Peptide sequence	Ratio of sialoglycosite abundance in 1,3,4-O-Bu_3ManNAc-treated to control cells: MCF10A	T-47D	MDA-MB-231
**6228[a] (1[b])**	YHY**NCS**LLDGTQLFTSHDYGAPQEATLGANK	7.14	2.42	5.80
**6229 (2)**	YHY**NGS**LMDGTLFDSSYSR	1.45	2.04	2.09
**6233 (3)**	YHY**NGT**LLDGTSFDTSYSK	18.93	1.80	1.78
**3971 (4)**	**NHT**YNTYIGQGYIIPGMDQGLQGACMGER	3.60	0.68	1.28
**6230 (5)**	YHY**NGT**FEDGK	1.04	0.09	0.16

[a] The data ID number is provided in **Column A** of **Sheet 1** of **Supplemental File #3**

[b] The glycosite # represents the labeling scheme used in **Figure 4** of the main text

Amino acid sequence (*from* Uniprot; https://www.uniprot.org/uniprot/Q96AY3)

N-glycan consensus sequons in **red** are predicted by NetNGly to be occupied

N-glycan consensus sequons in **blue** are predicted by NetNGly to be NOT occupied

Sequences underlined using a dotted line represent peptide sequences identified as "glycosites"

```
        10         20         30         40         50
MFPAGPPSHS LLRLPLLQLL LLVVQAVGRG LGRASPAGGP LEDVVIERYH
        60         70         80         90        100
IPRACPREVQ MGDFVRYHYN GTFEDGKKFD SSYDRNTLVA IVVGVGRLIT   ← Glycosite 6230 (5) [@N70]
       110        120        130        140        150
GMDRGLMGMC VNERRRLIVP PHLGYGSIGL AGLIPPDATL YFDVVLLDVW
       160        170        180        190        200
NKEDTVQVST LLRPPHCPRM VQDGDFVRYH YNGTLLDGTS FDTSYSKGGT   ← Glycosite 6233 (3) [@N182]
       210        220        230        240        250
YDTYVGSGWL IKGMDQGLLG MCPGERRKII IPPFLAYGEK GYGTVIPPQA
       260        270        280        290        300
SLVFHVLLID VHNPKDAVQL ETLELPPGCV RRAGAGDFMR YHYNGSLMDG   ← Glycosite 6229 (2) [@N294]
       310        320        330        340        350
TLFDSSYSRN HTYNTYIGQG YIIPGMDQGL QGACMGERRR ITIPPHLAYG   ← Glycosite 3971 (4) [@N310]
       360        370        380        390        400
ENGTGDKIPG SAVLIFNVHV IDFHNPADVV EIRTLSRPSE TCNETTKLGD
       410        420        430        440        450
FVRYHYNCSL LDGTQLFTSH DYGAPQEATL GANKVIEGLD TGLQGMCVGE   ← Glycosite 6228 (1) [@N407]
       460        470        480        490        500
RRQLIVPPHL AHGESGARGV PGSAVLLFEV ELVSREDGLP TGYLFVWHKD
       510        520        530        540        550
PPANLFEDMD LNKDGEVPPE EFSTFIKAQV SEGKGRLMPG QDPEKTIGDM
       560        570        580
FQNQDRNQDG KITVDELKLK SDEDEERVHE EL
```

TXNDC11 (Thioredoxin domain-containing protein 11)

Data ID[a] (Glycosite #)[b]	Peptide sequence	Ratio of sialoglycosite abundance in 1,3,4-O-Bu_3ManNAc-treated to control cells: MCF10A	T-47D	MDA-MB-231
**6278 (1)**	YLFPEVDMTST**NFT**GLSCR	0.08	1.24	1.42
**1317 (2)**	EVL**NYT**AENICK	0.04	25.07	1.17
**2125 (3)**	HF**NTS**LVFPR	1.24	1.96	0.86

[a] The data ID number is provided in **Column A** of **Sheet 1** of **Supplemental File #3**

[b] The glycosite # represents the labeling scheme used in **Figure 4** of the main text

Amino acid sequence (*from* Uniprot; https://www.uniprot.org/uniprot/Q6PKC3)

N-glycan consensus sequons in **red** are predicted by NetNGly to be occupied

N-glycan consensus sequons in **blue** are predicted by NetNGly to be NOT occupied

Sequences underlined using a dotted line represent peptide sequences identified as “glycosites”

```
        10         20         30         40         50
MSECGGRGGG SSSSEDAEDE GGGGGGPAGS DCLSSSPTLA TASSAGRLRR
        60         70         80         90        100
GLRGAFLMAR QRPELLCGAV ALGCALLLAL KFTCSRAKDV IIPAKPPVSF
       110        120        130        140        150
FSLRSPVLDL FQGQLDYAEY VRRDSEVVLL FFYAPWCGQS IAARAEIEQA
       160        170        180        190        200
ASRLSDQVLF VAINCWWNQG KCRKQKHFFY FPVIYLYHRS FGPIEYKGPM
       210        220        230        240        250
SAVYIEKFVR RVMKPLLYIP SQSELLDFLS NYEPGVLGYF EFSGSPQPPG
       260        270        280        290        300
YLTFFTSALH SLKKALESTS SPRALVSFTG EWHLETKIYV LDYLGTVRFG
       310        320        330        340        350
VITNKHLAKL VSLVHSGSVY LHRHFNTSLV FPR EVLNYTA ENICKWALEN   ← Glycosite 2125 (3) [@N326]
       360        370        380        390        400
QETLFRWLRP HGGKSLLLNN ELKKGPALFL FIPFNPLAES HPLIDEITEV   ← Glycosite 1317 (2) [@N337]
       410        420        430        440        450
ALEYNNCHGD QVVERLLQHL RRVDAPVLES LALEVPAQLP DPPTITASPC
       460        470        480        490        500
CNTVVLPQWH SFSRTHNVCE LCVNQTSGGM KPSSVSVPQC SFFEMAAALD
       510        520        530        540        550
SFYLKEQTFY HVASDSIECS NFLTSYSPFS YYTACCRTIS RGVSGFIDSE
       560        570        580        590        600
QGVFEAPTVA FSSLEKKCEV DAPSSVPHIE ENRYLFPEVD MTSTNFTGLS   ← Glycosite 6278 (1) [@N595]
       610        620        630        640        650
CRTNKTLNIY LLDSNLFWLY AERLGAPSST QVKEFAAIVD VKEESHYILD
       660        670        680        690        700
PKQALMKLTL ESFIQNFSVL YSPLKRHLIG SGSAQFPSQH LITEVTTDTF
       710        720        730        740        750
WEVVLQKQDV LLLYYAPWCG FCPSLNHIFI QLARNLPMDT FTVARIDVSQ
       760        770        780        790        800
NDLPWEFMVD RLPTVLFFPC NRKDLSVKYP EDVPITLPNL LRFILHHSDP
       810        820        830        840        850
ASSPQNVANS PTKECLQSEA VLQRGHISHL EREIQKLRAE ISSLQRAQVQ
       860        870        880        890        900
VESQLSSARR DEHRLRQQQR ALEEQHSLLH AHSEQLQALY EQKTRELQEL
       910        920        930        940        950
ARKLQELADA SENLLTENTW LKILVATMER KLEGRDGAES LAAQREVHPK
       960        970        980
QPEPSATPQL PGSSPPPANV SATLVSERNK ENRTD
```

SORL1 (Sortilin-related receptor)

Data ID[a] (Glycosite #)[b]	Peptide sequence	Ratio of sialoglycosite abundance in 1,3,4-O-Bu$_3$ManNAc-treated to control cells: MCF10A	T-47D	MDA-MB-231
**4642[a] (1[b])**	SDEY**NCS**SGMCIR	2.48	1.46	1.87
**207 (2)**	AI**NQT**AVECTWTGPR	2.06	0.18	1.33
**5633 (3)**	VG**NLT**AHTSYEISAWAK	4.49	77.19	1.27
**3565 (4)**	LTIV**NSS**VLDRPR	1.04	1.95	1.00
**33 (5)**	AAS**NFT**EIK	4.93	0.37	0.99
**6066 (6)**	VYGQVSL**NDS**HNQMVVHWAGEK	1.40	2.99	0.73
**2079 (7)**	GYEIHMFDSAM**NIT**AYLG**NTT**DNFFK	2.35	2.42	0.73
**6221 (8)**	YHIIVQLG**NMS**K	2.05	2.41	0.67
**5552 (9)**	VEGLQGVYIATLI**NGS**MNEENMR	1.67	272.6	0.38
**3761 (10)**	MGH**NYT**FTVQAR	0.67	2.44	0.22

[a] The data ID number is provided in **Column A** of **Sheet 1** of **Supplemental File #3**

[b] The glycosite # represents the labeling scheme used in **Figure 4** of the main text

Amino acid sequence (*from* Uniprot; https://www.uniprot.org/uniprot/Q92673)

N-glycan consensus sequons in **red** are predicted by NetNGly to be occupied

N-glycan consensus sequons in **blue** are predicted by NetNGly to be NOT occupied

Sequences underlined using a dotted line represent peptide sequences identified as "glycosites"

```
        10         20         30         40         50
MATRSSRRES RLPFLFTLVA LLPPGALCEV WTQRLHGGSA PLPQDRGFLV
        60         70         80         90        100
VQGDPRELRL WARGDARGAS RADEKPLRRK RSAALQPEPI KVYGQVSLND   ← Glycosite 6066 (6) [@N99]
       110        120        130        140        150
SHNQMVVHWA GEKSNVIVAL ARDSLALARP KSSDVYVSYD YGKSFKKISD
       160        170        180        190        200
KLNFGLGNRS EAVIAQFYHS PADNKRYIFA DAYAQYLWIT FDFCNTLQGF
       210        220        230        240        250
SIPFRAADLL LHSKASNLLL GFDRSHPNKQ LWKSDDFGQT WIMIQEHVKS
       260        270        280        290        300
FSWGIDPYDK PNTIYIERHE PSGYSTVFRS TDFFQSRENQ EVILEEVRDF
       310        320        330        340        350
QLRDKYMFAT KVVHLLGSEQ QSSVQLWVSF GRKPMRAAQF VTRHPINEYY
       360        370        380        390        400
IADASEDQVF VCVSHSNNRT NLYISEAEGL KFSLSLENVL YYSPGGAGSD
       410        420        430        440        450
TLVRYFANEP FADFHRVEGL QGVYIATLIN GSMNEENMRS VITFDKGGTW   ← Glycosite 5552 (9) [@N430]
       460        470        480        490        500
EFLQAPAFTG YGEKINCELS QGCSLHLAQR LSQLLNLQLR RMPILSKESA
       510        520        530        540        550
PGLIIATGSV GKNLASKTNV YISSSAGARW REALPGPHYY TWGDHGGIIT
       560        570        580        590        600
AIAQGMETNE LKYSTNEGET WKTFIFSEKP VFVYGLLTEP GEKSTVFTIF
       610        620        630        640        650
GSNKENVHSW LILQVNATDA LGVPCTENDY KLWSPSDERG NECLLGHKTV
       660        670        680        690        700
FKRRTPHATC FNGEDFDRPV VVSNCSCTRE DYECDFGFKM SEDLSLEVCV
       710        720        730        740        750
PDPEFSGKSY SPPVPCPVGS TYRRTRGYRK ISGDTCSGGD VEARLEGELV
       760        770        780        790        800
PCPLAEENEF ILYAVRKSIY RYDLASGATE QLPLTGLRAA VALDFDYEHN
       810        820        830        840        850
CLYWSDLALD VIQRLCLNGS TGQEVIINSG LETVEALAFE PLSQLLYWVD
       860        870        880        890        900
AGFKKIEVAN PDGDFRLTIV NSSVLDRPRA LVLVPQEGVM FWTDWGDLKP   ← Glycosite 3564 (4) [@N871]
       910        920        930        940        950
GIYRSNMDGS AAYHLVSEDV KWPNGISVDD QWIYWTDAYL ECIERITFSG
       960        970        980        990       1000
QQRSVILDNL PHPYAIAVFK NEIYWDDWSQ LSIFRASKYS GSQMEILANQ
      1010       1020       1030       1040       1050
LTGLMDMKIF YKGKNTGSNA CVPRPCSLLC LPKANNSRSC RCPEDVSSSV
      1060       1070       1080       1090       1100
LPSGDLMCDC PQGYQLKNNT CVKQENTCLR NQYRCSNGNC INSIWWCDFD
      1110       1120       1130       1140       1150
NDCGDMSDER NCPTTICDLD TQFRCQESGT CIPLSYKCDL EDDCGDNSDE
      1160       1170       1180       1190       1200
SHCEMHQCRS DEYNCSSGMC IRSSWVCDGD NDCRDWSDEA NCTAIYHTCE   ← Glycosite 4612 (1) [@N1164]
      1210       1220       1230       1240       1250
ASNFQCRNGH CIPQRWACDG DTDCQDGSDE DPVNCEKKCN GFRCPNGTCI
      1260       1270       1280       1290       1300
PSSKHCDGLR DCSDGSDEQH CEPLCTHFMD FVCKNRQQCL FHSMVCDGII
      1310       1320       1330       1340       1350
QCRDGSDEDA AFAGCSQDPE FHKVCDEFGF QCQNGVCISL IWKCDGMDDC
      1360       1370       1380       1390       1400
GDYSDEANCE NPTEAPNCSR YFQFRCENGH CIPNRWKCDR ENDCGDWSDE
      1410       1420       1430       1440       1450
KDCGDSHILP FSTPGPSTCL PNYYRCSSGT CVMDTWVCDG YRDCADGSDE
      1460       1470       1480       1490       1500
EACPLLANVT AASTPTQLGR CDRFEFECHQ PKTCIPNWKR CDGHQDCQDG
      1510       1520       1530       1540       1550
RDEANCPTHS TLTCMSREFQ CEDGEACIVL SERCDGFLDC SDESDEKACS
      1560       1570       1580       1590       1600
DELTVYKVQN LQWTADFSGD VTLTWMRPKK MPSASCVYNV YYRVVGESIW
      1610       1620       1630       1640       1650
KTLETHSNKT NTVLKVLKPD TTYQVKVQVQ CLSKAHNTND FVTLRTPEGL
      1660       1670       1680       1690       1700
PDAPRNLQLS LPREAEGVIV GHWAPPIHTH GLIREYIVEY SRSGSKMWAS
      1710       1720       1730       1740       1750
QRAASNFTEI KNLLVNTLYT VRVAAVTSRG IGNWSDSKSI TTIKGKVIPP   ← Glycosite 33 (5) [@N1706)
      1760       1770       1780       1790       1800
PDIHIDSYGE NYLSFTLTME SDIKVNGYVV NLFWAFDTHK QERRTLNFRG
      1810       1820       1830       1840       1850
SILSHKVGNL TAHTSYEISA WAKTDLGDSP LAFEHVMTRG VRPPAPSLKA   ← Glycosite 5633 (3)
      1860       1870       1880       1890       1900
KAINQTAVEC TWTGPRNVVY GIFYATSFLD LYRNPKSLTT SLHNKTVIVS
      1910       1920       1930       1940       1950
KDEQYLFLVR VVVPYQGPSS DYVVVKMIPD SRLPPRHLHV VHTGKTSVVI
      1960       1970       1980       1990       2000
KWESPYDSPD QDLLYAVAVK DLIRKTDRSY KVKSRNSTVE YTLNKLEPGG
      2010       2020       2030       2040       2050
KYHIIVQLGN MSKDSSIKIT TVSLSAPDAL KIITENDHVL LFWKSLALKE   ← Glycosite 6221 (8) @N2010]
      2060       2070       2080       2090       2100
KHFNESRGYE IHMFDSAMNI TAYLGNTTDN FFKISNLKMG HNYTFTVQAR   ← Glycosite 2079 (7) [two sites @N2069 and N2076]
                                                         ← Glycosite 3761 (10) [@N2092]
      2110       2120       2130       2140       2150
CLFGNQICGE PAILLYDELG SGADASATQA ARSTDVAAVV VPILFLILLS
      2160       2170       2180       2190       2200
LGVGFAILYT KHRRLQSSFT AFANSHYSSR LGSAIFSSGD DLGEDDEDAP
      2210
MITGFSDDVP MVIA
```

ST3GAL1 (CMP-N-acetylneuraminate-β-galactosamide-α-2,3-sialyltransferase 1)

Data ID[a] (Glycosite #)[b]	Peptide sequence	MCF10A	T-47D	MDA-MB-231
		Ratio of sialoglycosite abundance in 1,3,4-O-Bu_3ManNAc-treated to control cells:		
**1566[a] (1)[b]**	F**NQT**MQPLLTAQNALLEDDTYR	5.48	2.19	2.03
**5164 (2)**	TGVHDADFES**NVT**ATLASINK	0.95	2.54	1.47
**1156 (3)**	ELGD**NVS**MILVPFK	1.95	2.05	0.97

[a] The data ID number is provided in **Column A** of **Sheet 1** of **Supplemental File #3**

[b] The glycosite # represents the labeling scheme used in **Figure 5** of the main text

Amino acid sequence (*from* Uniprot; https://www.uniprot.org/uniprot/ Q11201)

N-glycan consensus sequons in **red** are predicted by NetNGly to be occupied

N-glycan consensus sequons in **blue** are predicted by NetNGly to be NOT occupied

Sequences underlined using a dotted line represent peptide sequences identified as "glycosites"

```
        10         20         30         40         50
MVTLRKRTLK VLTFLVLFIF LTSFFLNYSH TMVATTWFPK QMVLELSENL
        60         70         80         90        100
KRLIKHRPCT CTHCIGQRKL SAWFDERFNQ TMQPLLTAQN ALLEDDTYRW
       110        120        130        140        150
WLRLQREKKP NNLNDTIKEL FRVVPGNVDP MLEKRSVGCR RCAVVGNSGN
       160        170        180        190        200
LRESSYGPEI DSHDFVLRMN KAPTAGFEAD VGTKTTHHLV YPESFRELGD
       210        220        230        240        250
NVSMILVPFK TIDLEWVVSA ITTGTISHTY IPVPAKIRVK QDKILIYHPA
       260        270        280        290        300
FIKYVFDNWL QGHGRYPSTG ILSVIFSMHV CDEVDLYGFG ADSKGNWHHY
       310        320        330        340
WENNPSAGAF RKTGVHDADF ESNVTATLAS INKIRIFKGR
```

← **Glycosite 1566 (1)** [@N79]

← **Glycosite 5164 (2)** [@N201]

← **Glycosite 1156 (3)** [@N323]

ST3GAL4 (CMP-N-acetylneuraminate-β-galactosamide-α-2,3-sialyltransferase 4)

Data ID[a] (Glycosite #)[b]	Peptide sequence	Ratio of sialoglycosite abundance in 1,3,4-O-Bu_3ManNAc-treated to control cells: MCF10A	T-47D	MDA-MB-231
**3487[a] (1)[b]**	LR**NSS**LGDAINK	1.34	1.66	0.85
**3016 (2)**	LFG**NYS**R	1.47	0.39	0.45

[a] The data ID number is provided in **Column A** of **Sheet 1** of **Supplemental File #3**

[b] The glycosite # represents the labeling scheme used in **Figure 5** of the main text

Amino acid sequence (*from* Uniprot; https://www.uniprot.org/uniprot/ Q11206)

N-glycan consensus sequons in **red** are predicted by NetNGly to be occupied

N-glycan consensus sequons in **blue** are predicted by NetNGly to be NOT occupied

Sequences underlined using a dotted line represent peptide sequences identified as "glycosites"

```
        10         20         30         40         50
MVSKSRWKLL AMLALVLVVM VWYSISREDR YIELFYFPIP EKKEPCLQGE
        60         70         80         90        100
AESKASKLFG NYSRDQPIFL RLEDYFWVKT PSAYELPYGT KGSEDLLLRV   ← Glycosite 3487 (1) [@N61]
       110        120        130        140        150
LAITSSSIPK NIQSLRCRRC VVVGNGHRLR NSSLGDAINK YDVVIRLNNA   ← Glycosite 3016 (2) [@N131]
       160        170        180        190        200
PVAGYEGDVG SKTTMRLFYP ESAHFDPKVE NNPDTLLVLV AFKAMDFHWI
       210        220        230        240        250
ETILSDKKRV RKGFWKQPPL IWDVNPKQIR ILNPFFMEIA ADKLLSLPMQ
       260        270        280        290        300
QPRKIKQKPT TGLLAITLAL HLCDLVHIAG FGYPDAYNKK QTIHYYEQIT
       310        320        330
LKSMAGSGHN VSQEALAIKR MLEMGAIKNL TSF
```

ST6GAL1 (β-Galactoside α-2,6-sialyltransferase 1)

Data ID[a] (Glycosite #)[b]	Peptide sequence	MCF10A	T-47D	MDA-MB-231
		Ratio of sialoglycosite abundance in 1,3,4-O-Bu_3ManNAc-treated to control cells:		
**714[a] (1)[b]**	DHV**NVS**MVEVTDFPF**NTS**EWEGYLPK	0.60	619.47	0.98

[a] The data ID number is provided in **Column A** of **Sheet 1** of **Supplemental File #3**

[b] The glycosite # represents the labeling scheme used in **Figure 5** of the main text

Amino acid sequence (*from* Uniprot; https://www.uniprot.org/uniprot/ P15907)

N-glycan consensus sequons in **red** are predicted by NetNGly to be occupied

N-glycan consensus sequons in **blue** are predicted by NetNGly to be NOT occupied

Sequences underlined using a dotted line represent peptide sequences identified as "glycosites"

```
        10         20         30         40         50
MIHTNLKKKF SCCVLVFLLF AVICVWKEKK KGSYYDSFKL QTKEFQVLKS
        60         70         80         90        100
LGKLAMGSDS QSVSSSSTQD PHRGRQTLGS LRGLAKAKPE ASFQVWNKDS
       110        120        130        140        150
SSKNLIPRLQ KIWKNYLSMN KYKVSYKGPG PGIKFSAEAL RCHLRDHVNV
       160        170        180        190        200
SMVEVTDFPF NTSEWEGYLP KESIRTKAGP WGRCAVVSSA GSLKSSQLGR
       210        220        230        240        250
EIDDHDAVLR FNGAPTANFQ QDVGTKTTIR LMNSQLVTTE KRFLKDSLYN
       260        270        280        290        300
EGILIVWDPS VYHSDIPKWY QNPDYNFFNN YKTYRKLHPN QPFYILKPQM
       310        320        330        340        350
PWELWDILQE ISPEEIQPNP PSSGMLGIII MMTLCDQVDI YEFLPSKRKT
       360        370        380        390        400
DVCYYYQKFF DSACTMGAYH PLLYEKNLVK HLNQGTDEDI YLLGKATLPG

FRTIHC
```

Glycosite 714 (1) [@N149 and N161]

(the peptide contains both predicted sites of N-glycosylation)

ST6GALNAC2 (α-N-Acetylgalactosaminide α-2,6-sialyltransferase 2)

Data ID[a] (Glycosite #)[b]	Peptide sequence	Ratio of sialoglycosite abundance in 1,3,4-O-Bu_3ManNAc-treated to control cells: MCF10A	T-47D	MDA-MB-231
**1895[a] (1)[b]**	GLSHQVIASTLSLL**NGS**ESAK	0.08	0.10	4.52

[a] The data ID number is provided in **Column A** of **Sheet 1** of **Supplemental File #3**

[b] The glycosite # represents the labeling scheme used in **Figure 5** of the main text

Amino acid sequence (*from* Uniprot; https://www.uniprot.org/uniprot/ Q9UJ37)

N-glycan consensus sequons in **red** are predicted by NetNGly to be occupied

N-glycan consensus sequons in **blue** are predicted by NetNGly to be NOT occupied

Sequences underlined using a dotted line represent peptide sequences identified as "glycosites"

```
        10         20         30         40         50
MGLPRGSFFW LLLLLTAACS GLLFALYFSA VQRYPGPAAG ARDTTSFEAF
        60         70         80         90        100
FQSKASNSWT GKGQACRHLL HLAIQRHPHF RGLFNLSIPV LLWGDLFTPA
       110        120        130        140        150
LWDRLSQHKA PYGWRGLSHQ VIASTLSLLN GSESAKLFAP PRDTPPKCIR   <- Glycosite 1895 (1) [@N130]
       160        170        180        190        200
CAVVGNGGIL NGSRQGPNID AHDYVFRLNG AVIKGFERDV GTKTSFYGFT
       210        220        230        240        250
VNTMKNSLVS YWNLGFTSVP QGQDLQYIFI PSDIRDYVML RSAILGVPVP
       260        270        280        290        300
EGLDKGDRPH AYFGPEASAS KFKLLHPDFI SYLTERFLKS KLINTHFGDL
       310        320        330        340        350
YMPSTGALML LTALHTCDQV SAYGFITSNY WKFSDHYFER KMKPLIFYAN
       360        370
HDLSLEAALW RDLHKAGILQ LYQR
```

ST8SIA4 (CMP-N-acetylneuraminate-poly-α-2,8-sialyltransferase)

Data ID[a] (Glycosite #)[b]	Peptide sequence	Ratio of sialoglycosite abundance in 1,3,4-O-Bu_3ManNAc-treated to control cells: MCF10A	T-47D	MDA-MB-231
**2556[a] (1)[b]**	I**NSS**LVLEIR	3.53	1.42	0.84

[a] The data ID number is provided in **Column A** of **Sheet 1** of **Supplemental File #3**

[b] The glycosite # represents the labeling scheme used in **Figure 5** of the main text

Amino acid sequence (*from* Uniprot; https://www.uniprot.org/uniprot/ 92187)

N-glycan consensus sequons in **red** are predicted by NetNGly to be occupied

N-glycan consensus sequons in **blue** are predicted by NetNGly to be NOT occupied

Sequences underlined using a dotted line represent peptide sequences identified as "glycosites"

```
        10         20         30         40         50
MRSIRKRWTI CTISLLLIFY KTKEIARTEE HQETQLIGDG ELSLSRSLVN
        60         70         80         90        100
SSDKIIRKAG SSIFQHNVEG WKINSSLVLE IRKNILRFLD AERDVSVVKS   <- Glycosite 2556 (1) [@N74]
       110        120        130        140        150
SFKPGDVIHY VLDRRRTLNI SHDLHSLLPE VSPMKNRRFK TCAVVGNSGI
       160        170        180        190        200
LLDSECGKEI DSHNFVIRCN LAPVVEFAAD VGTKSDFITM NPSVVQRAFG
       210        220        230        240        250
GFRNESDREK FVHRLSMLND SVLWIPAFMV KGGEKHVEWV NALILKNKLK
       260        270        280        290        300
VRTAYPSLRL IHAVRGYWLT NKVPIKRPST GLLMYTLATR FCDEIHLYGF
       310        320        330        340        350
WPFPKDLNGK AVKYHYYDDL KYRYFSNASP HRMPLEFKTL NVLHNRGALK

LTTGKCVKQ
```

NEU1 (Sialidase 1)

Data ID[a] (Glycosite #)[b]	Peptide sequence	Ratio of sialoglycosite abundance in 1,3,4-O-Bu_3ManNAc-treated to control cells: MCF10A	T-47D	MDA-MB-231
**4071[a] (1)[b]**	**NLS**LDIGTEVFAPGPGSGIQK	8.69	1.18	15.23
**6120 (2)**	WSFS**NGT**SWR	1.08	2.21	1.07

[a] The data ID number is provided in **Column A** of **Sheet 1** of **Supplemental File #3**

[b] The glycosite # represents the labeling scheme used in **Figure 5** of the main text

Amino acid sequence (*from* Uniprot; https://www.uniprot.org/uniprot/ Q99519)

N-glycan consensus sequons in **red** are predicted by NetNGly to be occupied

N-glycan consensus sequons in **blue** are predicted by NetNGly to be NOT occupied

Sequences underlined using a dotted line represent peptide sequences identified as "glycosites"

```
        10         20         30         40         50
MTGERPSTAL PDRRWGPRIL GFWGGCRVWV FAAIFLLLSL AASWSKAEND
        60         70         80         90        100
FGLVQPLVTM EQLLWVSGRQ IGSVDTFRIP LITATPRGTL LAFAEARKMS
       110        120        130        140        150
SSDEGAKFIA LRRSMDQGST WSPTAFIVND GDVPDGLNLG AVVSDVETGV
       160        170        180        190        200
VFLFYSLCAH KAGCQVASTM LVWSKDDGVS WSTPRNLSLD IGTEVFAPGP  ← Glycosite 4071 (1) [@N186]
       210        220        230        240        250
GSGIQKQREP RKGRLIVCGH GTLERDGVFC LLSDDHGASW RYGSGVSGIP
       260        270        280        290        300
YGQPKQENDF NPDECQPYEL PDGSVVINAR NQNNYHCHCR IVLRSYDACD
       310        320        330        340        350
TLRPRDVTFD PELVDPVVAA GAVVTSSGIV FFSNPAHPEF RVNLTLRWSF
       360        370        380        390        400
SNGTSWRKET VQLWPGPSGY SSLATLEGSM DGEEQAPQLY VLYEKGRNHY  ← Glycosite 6120 (2) [@N352]
       410
TESISVAKIS VYGTL
```
